# Classification of ECG signals using multi-cumulants based evolutionary hybrid classifier

**DOI:** 10.1038/s41598-021-94363-6

**Published:** 2021-07-23

**Authors:** Sahil Dalal, Virendra P. Vishwakarma

**Affiliations:** University School of Information, Communication and Technology, Guru Gobind Singh Indraprastha University, Sector 16-C, Dwarka, New Delhi, India

**Keywords:** Biomedical engineering, Computer science, Arrhythmias

## Abstract

Every human being has a different electro-cardio-graphy (ECG) waveform that provides information about the well being of a human heart. Therefore, ECG waveform can be used as an effective identification measure in biometrics and many such applications of human identification. To achieve fast and accurate identification of human beings using ECG signals, a novel robust approach has been introduced here. The databases of ECG utilized during the experimentation are MLII, UCI repository arrhythmia and PTBDB databases. All these databases are imbalanced; hence, resampling techniques are helpful in making the databases balanced. Noise removal is performed with discrete wavelet transform (DWT) and features are obtained with multi-cumulants. This approach is mainly based on features extracted from the ECG data in terms of multi-cumulants. The multi-cumulants feature based ECG data is classified using kernel extreme learning machine (KELM). The parameters of multi-cumulants and KELM are optimized using genetic algorithm (GA). Excellent classification rate is achieved with 100% accuracy on MLII and UCI repository arrhythmia databases, and 99.57% on PTBDB database. Comparison with existing state-of-art approaches has also been performed to prove the efficacy of the proposed approach. Here, the process of classification in the proposed approach is named as evolutionary hybrid classifier.

## Introduction

**Figure 1:**
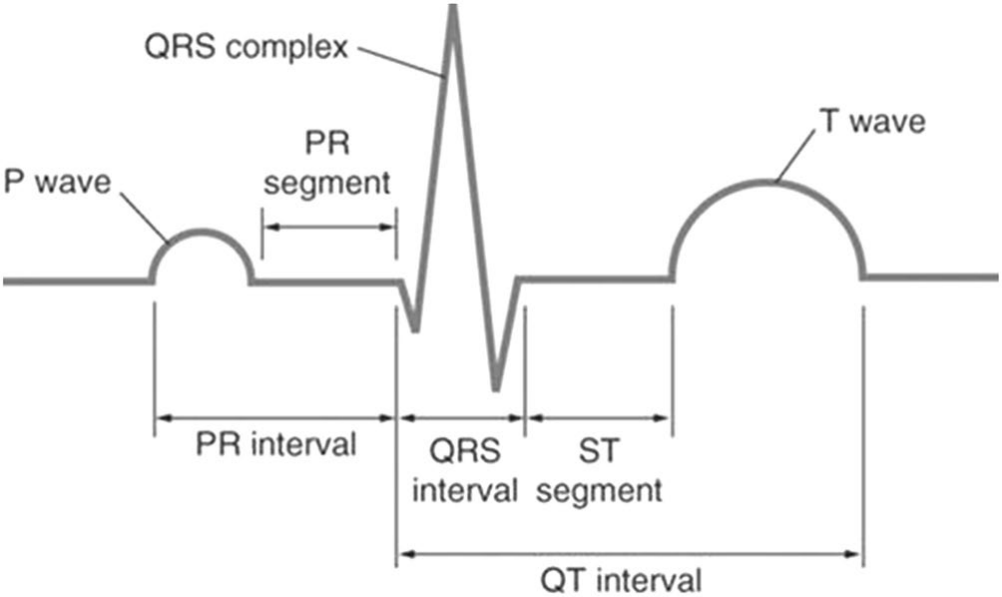
Standard ECG waveform.

Among non-linear signal analysis, Electro-Cardio-Graphy (ECG) is a signal of quite an interest for the researchers since last many decades. This is because ECG is quite common in modelling the biometric systems. Authentication methods utilized traditionally were based on fingerprints and face recognition. These methods have become susceptible to falsification. ECG can be a best-fit for biometric systems because of its advantages like uniqueness of ECG for every person. Moreover, ECG recording can only be possible in living things and hence, it is difficult to forge. Therefore, differentiating one ECG from the other can be very helpful. Also, it plays an important role in the prevention of cardiovascular diseases by providing a diagnostic measure. A standard ECG waveform is a recording of the electrical activity of the heart consisting of P, Q, R, S and T wave as shown in Fig. 1. Since ECG has many applications in various fields like medical^1^, Internet of Things, cryptography, wearable sensors^2,3^ etc., thus, researchers have done enormous amount of research work on ECG as given in a comparative analysis on ECG by Ikenna Odinaka et al.^4^.

It all started in 1981 when Leif Sörnmo et al*.* introduced a method to evaluate the QRS-complex features using the mathematical modelling of Euclidean distance of similar complex class^5^. This can also be performed using linear prediction as proven by Lin and Chang^6^. Then, in 1996, a non-deterministic and continuous probability density function model is introduced for this non-linear signal recognition. The hidden markov model was earlier used in DNA modelling and speech recognition which are also non-linear signals and hence, were best suited for ECG signals^7^. Giovanni Bortolan et al. gave the possibilities of using neural networks in classifying the ECG signals^8^. Neural network was further implemented in the next year by Zümray Dokur et al. for ECG signals recognition when peaks of QRS complex in the ECG waveform were utilized as feature vectors obtained from the DFT spectrum^9^. This work of feature extractor is extended when Botter et al. exploited the asymmetric basis functions for extracting features from peaks of M shaped P waves in ECG signals^10^. Neural network is a learn and train network and widely used in ECG signals analysis. In 1998, Ischemia detection was introduced in ECG investigation, by T. Stamkopoulos et al., when principal component analysis (PCA) was used with neural network. Ischemia is an ECG waveform having a small positive J peak between ST segments. The method was trained with 1000 samples of ST-T database and achieved 90% of recognition rate in detecting ischemia^11^. Also, in 1999, Z. Dokur et al. gave a comparison on Fourier transform and discrete wavelet transform while classifying ECG beats^12^. The concept of designing a mathematical modelling relatable to the real world applications is also helpful in interpreting an ECG Signal. It came into existence when M. Kundu et al. introduced the concept of fuzzy logic in ECG interpretation^13^. This concept of fuzzy logic was again utilized when extended version i.e. type-2 fuzzy clustering was implemented with wavelet transform and neural network for the classification of ECG signals^14^. In 2001, Lena Biel et al. experimented and concluded that one lead can also be helpful in extracting features from ECG and recognize a person^15^. Mohamed I. Owis et al*.* proposed and reported a model using features obtained from Lyapunov exponent and correlation dimension for the successful detection and classification of ECG signals^16^. An ECG signal coder, having low computational complexity, was designed by applying N-PR cosine modulated filter banks to have low bit rate in 2004^17^. M.G. Tsipouras et al*.* exploited the RR interval in ECG waveforms for the beat and episode classification by achieving 98% and 94% of accuracy respectively^18^. ECG is also helpful in identifying an individual. It was done in 2005 when Steven A. Israel et al*.* computed the features using the fiducial points obtained from filtered data of ECG and characterize the uniqueness of an individual^19^. The work of knowledge-based ECG interpretation was further extended and rule-based rough-set based ECG classification was generated by S. Mitra in 2006. They had introduced an offline system for ECG data acquisition which produced noisy data. Therefore, proper noise removal and baseline correction was performed before applying the proposed method. Respective peaks in the ECG waveforms were detected for the classification^20^. A method for noise removal was also proposed by B. N. Singh and A. K. Tiwari in 2006 which utilized mother wavelet basis function for denoising of ECG signals while retaining the ECG peaks to the same as in noisy data^21^. In the same year, a comparative study was also proposed on ECG descriptors for the heartbeats classification. This was between morphological and time frequency descriptors. Morphological features include QRS pattern recognition while computing expansion coefficients using matching pursuits algorithm gave time frequency correlation. The heartbeats are taken from MIT-BIH arrhythmia database and four local sets of GLS and classified using*k* nearest neighbour classifier^22,23^. Very good accuracy was achieved with both the descriptors^24^. Yeong Pong Meau et al*.* introduced a novel technique for ECG classification in 2006. The technique was a hybrid of extended kalman filter and neuro fuzzy system and helpful in distinguishing various abnormal ECG signals. Due to the use of multi-layer perceptron network in neuro fuzzy system, the technique was iterative and hence, computational complexity is high^25^. In one more research, DWT was utilized to decompose the ECG into time and frequency domain to compute the wavelet coefficients and classification of ECG beats were performed using multiclass support vector machine ^26^. Independent component analysis was also implemented to decompose the ECG signals into weighted sum of basic components that are statistically mutual independent. This feature vector was formed by combining these components with the RR interval and classified using various classifiers like Bayes, minimum distance and neural network classifier^27,28^. These features of independent component analysis and RR interval when combined with wavelet transform features, 99.3% of accuracy was achieved using SVM on 16 classes of MIT-BIH databases^29^. In 2008, Argyro Kampouraki et al*.* utilized the statistical analysis for feature extraction of two ECG databases: young and elderly ECG signals & normal and abnormal ECG signals. The classification was performed using SVM with very low signal to noise ratio^30^. The parameters of SVM like Gaussian radial basis function (RBF) and penalty parameter were also optimized using genetic algorithm (GA) for ECG arrhythmias classification^31^. The authors also performed the same task by changing the optimization algorithm from GA to PSO giving better results than the earlier approach. PSO based optimization is faster as well compared to GA^32^. In medical applications, ECG was introduced for age classification by M. Wiggins et al*.* with the help of genetically optimized Bayesian classifier achieving 86.25% AUC which is better compared to other existing methods^1^. Turker Ince et al*.* proposed a method for ECG patterns recognition by applying wavelet transform for feature extraction and PCA for dimensionality reduction. The classification was performed using neural network optimized using particle swarm analysis (PSO). The method even achieved higher accuracy on larger databases^33^. PCA was also combined with linear discriminant analysis (LDA) for feature reduction and using probabilistic neural network classifier ECG arrhythmias were classified with 99.71% of accuracy^34^. In 2009, Walter Karlen et al*.* combined the fast fourier transform and artificial neural network for the sleep and wake states in ECG signals obtained with the help of wearable sensors. 86.7% of accuracy was achieved on multiclass data as satisfactory performance^2^. Sleep apnea was also detected by Baile Xie and Hlaing Minn in 2012 using saturation of peripheral oxygen ad combination of various classifiers^35^. Comparisons of DWT, continuous wavelet transform (CWT) and discrete cosine transform (DCT) were performed on Massachusetts Institute of Technology-Beth Israel Hospital (MIT-BIH) databases^36^ by using neural network and SVM by Hamid Khorrami and Majid Moavenian in 2010^37^. Then, Yüksel Özbay and Gülay Tezel introduced a neural network for ECG classification which has adaptive activation function. The results achieved over 92 patients of ECGs were 98.19% which is quite good^38,39^. A unique way of Teager energy function was firstly utilized for ECG beat classification by C. Kamath in 2011. The advantage of using Teager energy function for ECG was that this function models the energy of the source such that the activity in the heart can easily be visible in the function^40^. ECG recordings can also be used for the recognition of emotions in a person. It was introduced by Guo Xianhai by utilizing radial basis function neural network and achieved an accuracy of 91.67%^41^. Till 2015, hybrid of classifiers was exploited. It was in this year that K. Muthuvel et al*.* utilized the hybrid features for the classification of ECG beats. Morphological based features were combined with Haar wavelet features and tri spectrum features. The resultant features vectors were classified using feed forward neural network with 78% of accuracy achieved over MIT-BIH database^42^. E. Alickovic and A. Subasi utilized multiscale PCA and Autoregression (AR) modelling for designing a recognition method using various classifiers to diagnose heart diseases. 99.93% accuracy was achieved on MIT-BIH database^43^. In the next year, the authors had used RF classifiers for ECG signals classification with the help of decomposition of ECG signals using DWT^44^. Concept of approximate entropy was combined with wavelet decomposition in 2016 by Hongqiang Li et al*.* for ECG signal classification using SVM classifier. The algorithm was fast, simple in computation and achieved 97.78% of accuracy in five beats classification^45^. The author again applied the optimization algorithm on extracted features with GA in a scientific report and improved the accuracy to 99.33%^46^. Padmavathi Kora and K. Sri Rama Krishna proposed an approach in the same year when extracted feature from ECG signal was optimized using Bat algorithm. The classification was binary i.e. two classes normal and abnormal ECG which was performed with two hidden layers neural network^47^. An analysis of ECG signals was also performed using PCA and hybrid of neural network with fuzzy classifier. The Neuro-Fuzzy classifier came out with 95.83% of accurate results in classifying the ECGs into their respective classes^48^. Fuzzy C- means clustering combined with Mahalanobis Distance and utilized for arrhythmic beats classification. It was done to improve the improper clustering that was occurring because of spherical clusters detected with Euclidean distance based clustering. The method improved the results to a great extent^49^. Sibasankar Padhy and S. Dandapat introduced a technique for myocardial infrection classification in ECG signals. The technique utilized leads, beats and samples to represent the data in third order tensor structure. Higher order singular value decomposition and mode-*n* singular values were exploited as features and finally, classified using SVM with 95.30% accuracy on Physikalisch-Technische Bundesanstalt Database (PTBDB) classifier^50^. The concept of deep neural network (DNN)^51^ was also introduced in ECG signals classification like convolutional neural networks^52^, DNN using stacked denoising autoencoder or 1D CNN^53–55^. The 1-dimensional convolutional neural network (1D CNN) was exploited for classification of heart sound. Autoencoder was exploited for extraction of features and stated better results compared to back propagation neural network ^55,56^. In 2019, Mohamed Hammad et al*.* also utilized the concept of 12-layer CNN for the ECG signals classification with PTBDB database and achieved 98.37% of accuracy^57^. Leandro B. Marinho et al*.* gave an analysis based on various feature extraction techniques like Goertzel, structural co-occurance matrix, higher order statistics and Fourier transform. These individual features were classified using SVM, Bayesian, multi-layer perceptron and optimum path forest classifiers. The Combination of higher order statistics with Bayesian classifier gives the best result among these with 94.3% accuracy in classification^58^. In the same year, S. Velmurugan et al*.* introduced the Gabor wavelet transform with multi linear discriminant analysis to reduce the execution time in features extraction from ECG data of UCI repository Arrhythmia database^59,60^. Giansalvo Cirrincione et al*.* performed a comparative analysis for the extraction of features from the ECG signals using the neural network classifier with promising results. PCA was also utilized for dimension reduction^61^. Chandan Kumar Jha and Maheshkumar H. Kolekar proposed a technique using Q-wavelet decomposed to sixth level based features of MIT-BIH database and classified using SVM classifier with very good results in classifying the ECG beats^62^. S. Mian Qaisar and A. Subasi introduced an event driven ECG signal acquisition and achieved 94.07% accuracy on MIT-BIH database using machine learning techniques^63^. Paweł Pławiak proposed a technique named as evolutionary neural system in which MLII database of ECG was classified into 17 classes with SVM classifier and 98.85% of accuracy was achieved^64^. Özal Yildirim et al*.* also gave solution for MLII ECG database with deep convolutional neural network and 91.33% of accuracy was reported^65^.

### Motivation and contribution

Approaches available in literature pose various limitations like higher time complexity due to iterative nature of algorithms, requirement of more analysis for specific features and unbalanced databases etc. So, there should be a robust method that can overcome all these limitations and perform the ECG signals classification more effectively and efficiently. To give solutions for the aforementioned disadvantages of the existing techniques, a robust and novel approach is introduced. The approach utilizes technique for balancing of the ECG database used during experimentation. A technique for noise removal and baseline correction of ECG signals is also added in the approach. Feature extraction is performed with the help of a unique technique named as Multi-cumulants^66^ that has never been utilized in the ECG analysis. The features obtained from second-, third- and fourth-order cumulants are concatenated to form a feature vector. The feature vector is then used for classification. The classification is performed with a non-iterative method of machine learning. It is named as kernel extreme learning machine (KELM)^67^. The parameters of KELM are optimized using an optimization algorithm. The hybrid of KELM and optimization algorithm is termed, here, as evolutionary hybrid classifier.

The remaining paper is organised in the following manner: “Preliminaries” gives a brief idea about the preliminaries and databases used during the experimentation of the proposed approach. “Proposed method” tells about the proposed method of ECG analysis. “Experimental results and analyses” discusses the experimental results and analyses followed by conclusion in “Conclusion and future scope”.

## Preliminaries

**Figure 2:**
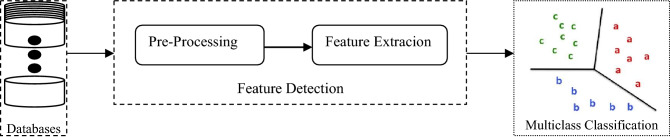
Basic overview of a general recognition system.

The proposed method for the recognition of ECG signals consists of three steps: pre-processing, feature extraction and classification. A basic overview of a general recognition system is shown in Fig. 2. Detailed block diagram is explained in the next section. Pre-processing step includes balancing of the ECG databases utilized during experimentation and noise filtering in the ECG signals which is performed with the help of resampling techniques and wavelet transform respectively. Feature extraction is done using cumulants and a feature vector is obtained as multi-cumulants features. Pre-processing and feature extraction stages have been combined and known as feature detection in this approach. At last, ECG signals are classified into their respective classes by using evolutionary hybrid classifier. A brief overview of all the preliminaries used is given as follows:

### Resampling techniques

First step towards pre-processing in the proposed approach is balancing of the database. Uneven number of samples in various classes of a database is quite common. However, it increases the chances of error during classification when the difference between numbers of samples in a class is very large compared to other classes. Therefore, the approach utilized for sample recognition becomes sensitive towards the class having major number of samples in the database. Thus, data balancing is very important in such uneven or unbalanced databases. Resampling techniques are those techniques which are used for the balancing of samples in each class of a database. There can be so many techniques for data balancing but commonly classified into over-sampling, under-sampling and hybrid of these two (known as importance resampling)^68^.

#### Random oversampling technique

**Figure 3:**
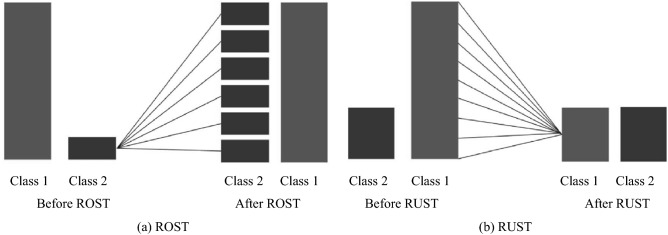
A sample showing resampling techniques on two class database.

Random OverSampling Technique (ROST) is a resampling technique utilized for balancing the unbalanced data in the database. It is a non recursive approach as it randomly copies the data of the class having less number of samples to make the samples equal in the respective class (the minor class) to the class (the major class) having highest number of samples in that database. It is shown in Fig. 3a for better understanding. As it can be seen from the figure, before applying ROST, Class 1 is the major class containing very large number of samples as compared to Class 2 (the minor class). ROST copies the samples of Class 2 randomly and makes itself equal to the Class 2. This technique of resampling is very effective in giving better results of recognition in machine learning because copying the same data into the classes helps in getting good training of the machine learning approach so that efficient model can be formed. Along with this advantage, ROST also results into over-fitting of the data which is a substantial drawback for the technique and is rectified at the classification stage of our proposed method.

#### Random undersampling technique (RUST)

In the next resampling technique, as the name states in itself, Random Under Sampling Technique (RUST) is the opposite of ROST. It reduces the number of samples in major class and makes them equal to the number of samples in minor classes. This is done randomly and hence, results in loss of important data from the samples. RUST is also explained in Fig. 3b. Here in Fig. 3b, before applying RUST, Class 2 is the minor class containing very less number of samples as compared to the Class 1 (the major class). RUST reduces the samples of Class 1 randomly and makes it equal to the samples in Class 2. Due to this reduction in the number of samples, results also affect in recognizing the sample.

#### Importance resampling technique

Importance ReSampling Technique (IRST) is a hybrid of ROST and RUST. IRST overcomes the limitations of ROST and RUST by combining the advantages of both of these techniques into one. This technique uses important or weighted information of the data and reframes the data according to their importance in the database. The weight acts as the carrier to the data and the most important is assigned with the weights to remove the least important data only.

As there is loss of enormous amount of data in RUST (as shown in Tables 1 and 2), therefore, only ROST and IRST have been utilized for balancing the number of samples in the classes of databases for the proposed approach. After balancing the data, noise removal is performed in the ECG signals using wavelet transform.

**Table 1:** Comparison of number of samples in the classes in balanced MLII ECG Database with number of samples in the classes in unbalanced MLII ECG database.

S. No	Class	Unbalanced database	ROST	IRST	RUST
1	Normal sinus rhythm (NSR)	283	283	59	10
2	Atrial premature beat (APB)	66	283	59	10
3	Atrial flutter (AFL)	20	283	59	10
4	Atrial fibrillation (AFIB)	135	283	59	10
5	Supraventricular tachyarrhythmia (SVTA)	13	283	59	10
6	Pre-excitation (WPW)	21	283	59	10
7	Premature ventricular contraction (PVC)	133	283	59	10
8	Ventricular bigeminy (BIG)	55	283	59	10
9	Ventricular trigeminy (TRI)	13	283	59	10
10	Ventricular tachycardia (VT)	10	283	59	10
11	Idioventricular rhythm (IVR)	10	283	59	10
12	Ventricular flutter (VFL)	10	283	59	10
13	Fusion of ventricular and normal beat (FUS)	11	283	59	10
14	Left bundle branch block beat (LBBBB)	103	283	59	10
15	Right bundle branch block beat (RBBBB)	62	283	59	10
16	Second-degree heart block (SDHB)	10	283	59	10
17	Pacemaker rhythm (PR)	45	283	59	10

**Table 2:** Comparison of number of samples in the classes in balanced PTBDB ECG database with number of samples in the classes in unbalanced PTBDB ECG database.

S.No	Class	Unbalanced Database	ROST	IRST	RUST
1	Normal	4046	10,506	7276	4046
2	Abnormal	10,506	10,506	7276	4046

### Wavelet transform

Wavelet transform (WT) takes its origin from Fourier transform. In Fourier transform, signals are transformed into frequency domain so that analysis of the ECG signal can be done easily. This is because computations in time domain are difficult as compared to the computations in frequency domain. For example, convolution in time domain is simple multiplication in the frequency domain. A general equatorial representation of WT (assuming finite energy and zero mean) is as follows:

1 $${W}_{T}\left(\Gamma, \sigma \right)= \frac{1}{\sqrt{\sigma }}\int_{t}x\left(t\right){\Phi }^{*}\left(\frac{t-\Gamma }{\sigma }\right)dt$$

Here, *W*_*T*_ represents the wavelet coefficients of convolution of the signal *x*(*t*) with mother wavelet function *Φ*(*t*). *Γ* is the measure of time known as translation and *σ* is the measure of frequency known as scaling parameters. By taking different combinations of *Γ* and *σ*, various mother wavelet functions can be generated. There can be different families of wavelet transforms and that are Haar, symmlet, coiflet, Daubechies, Mexican hat, B-splines, and many more.

In discrete time domain, discrete wavelet transforms are defined as:

2 $$A={z}_{low}\left[p\right]=\sum x\left[m\right].{r}_{h}[2p-m]$$

3 $$D={z}_{high}\left[p\right]=\sum x\left[m\right].{r}_{l}[2p-m]$$

It is nothing but the decomposition of the signal using successive filtering with the help of low and high pass filters. *A* is the approximation coefficient and *D* is the detailed coefficient. *A* and *D* are obtained using dyadic decomposition of signal using successive low pass and high pass filters respectively. *r*_*h*_ and *r*_*l*_ are the high and low pass filters in dyadic DWT with half the cut off frequency from the previous frequency.

**Figure 4:**
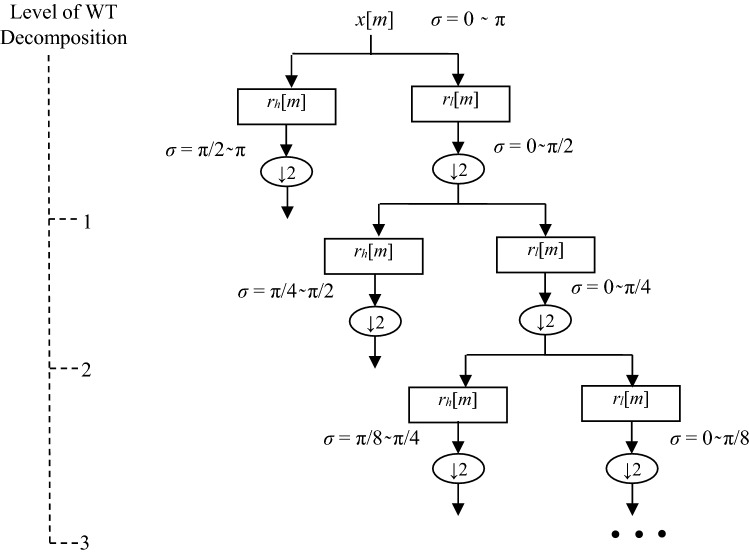
Decomposition of signal *x*[*n*] on the basis of WT.

The scaling and wavelet functions in discrete WT are represented mathematically as:

4 $${\Theta }_{m,n}\left(t\right)={2}^{m/2}.\Theta ({2}^{m}t-n)$$

5 $${\Phi }_{m,n}\left(t\right)={2}^{m/2}.\Phi ({2}^{m}t-n)$$

where *m,n ∈ Z*. Figure 4 shows the decomposition of a signal on the basis of WT.

WT provides a multi-resolution system. Signals having discontinuity have many coefficients with large magnitude using Fourier transform but WT generates few significant coefficients around the discontinuity and set the rest to zero. Hence, better results are achieved in non approximation with WT during reconstruction of the signals. Due to this advantage of WT, it is also helpful in achieving good accuracy results in compression and denoising of the signals. Therefore, WT is utilized here for the denoising of the ECG signals. Now, the features will be computed for these denoised signals and it will be performed with the help of Cumulants.

### Cumulants

Higher order statistics are stated in terms of moments (*n*_*m*_) and cumulants (*K*_*m*_). *K*_*m*_ are stated as the set of components that are generated using the non-linear combinations of moments^69^. Generating function *f*(*t*) is also helpful in defining the *K*_*m*_ and for a random variable *Y*, *f*(*t*) is represented as:

6 $$f\left(t\right)=logE[{e}^{tY}]$$

where *E* is the statistical expectation and defined for random variable *Y,* having probability distribution function *g*(*y*), as:

7 $$E\left[Y\right]={\int }_{-\infty }^{\infty }y.g\left(y\right)dy$$

Cumulants (*K*_*m*_) are obtained from the power series expansion of cumulant generating function and represented as:

8 $$f\left(t\right)=\sum_{m=1}^{\infty }{K}_{m}\frac{{t}^{m}}{m!}$$

Therefore, *K*_*m*_ can be defined as MacLaurin series expansion in which *m*th order Cumulant is calculated at *t* = 0.

9 $${K}_{m}={\left.\frac{{\partial }^{m}}{{\partial t}^{m}}f(t)\right|}_{t=0}$$

Now, Cumulants (*K*_*m*_) are represented using combinations of moments (*n*_*m*_). The moments (*n*_*m*_) existing for upto *l* order, say for a non-stationary signal *z* (*m*) which depends only on the time differences ${\epsilon }_{1},{\epsilon }_{2},\dots ,{\epsilon }_{l-1}$ where *m* = 0, ± 1, ± 2, ± 3, ± 4, ……, in terms of *E*, is

10 $${n}_{l}\left({\epsilon }_{1},{\epsilon }_{2},\dots ,{\epsilon }_{l-1}\right)=E\left[z\left(m\right).z\left(m+{\epsilon }_{1}\right)\dots z\left(m+{\epsilon }_{l-1}\right)\right]$$

Hence, using this equation, the first-order cumulant *K*_*1*_ is stated as

11 $${K}_{1}={n}_{1}=E[z\left(m\right)]$$

It is clearly visible that it is equal to the first-order moment and is defined as the mean value of the non-stationary signal *z* (*m*). Similarly, the second-order cumulant ${K}_{2}(\epsilon )$ is shown in the form of equation as:

12 $${K}_{2}\left(\epsilon \right)={n}_{2}\left(\epsilon \right)-{({n}_{1})}^{2}$$

Here, ${n}_{2}\left(\epsilon \right)$ is the second-order moment defined as the autocorrelation and ${K}_{2}\left(\epsilon \right)$ denotes the second-order cumulant that is called as variance. For zero mean variables, ${K}_{2}\left(\epsilon \right)={n}_{2}\left(\epsilon \right)$. The third-order cumulant ${K}_{3}\left({\epsilon }_{1},{\epsilon }_{2}\right)$ is represented as

13 $${K}_{3}\left({\epsilon }_{1},{\epsilon }_{2}\right)={n}_{3}\left({\epsilon }_{1},{\epsilon }_{2}\right)-{n}_{1}\left[{n}_{2}\left({\epsilon }_{1}\right)+{n}_{2}\left({\epsilon }_{2}\right)-{n}_{2}\left({\epsilon }_{1}-{\epsilon }_{2}\right)\right]+2{({n}_{1})}^{3}$$

In this, ${n}_{3}\left({\epsilon }_{1},{\epsilon }_{2}\right)$ depicts the third-order moment. ${K}_{3}\left({\epsilon }_{1},{\epsilon }_{2}\right)$ explains the skewness of the signal and is equal to ${n}_{3}\left({\epsilon }_{1},{\epsilon }_{2}\right)$ for zero-mean. When there is symmetric signal, ${K}_{3}\left({\epsilon }_{1},{\epsilon }_{2}\right)$ becomes zero and for zero-mean variables, cumulants are equal to moments upto third-order i.e. ${K}_{3}\left({\epsilon }_{1},{\epsilon }_{2}\right)={n}_{3}\left({\epsilon }_{1},{\epsilon }_{2}\right)$. Therefore, fourth-order cumulant ${K}_{4}\left({\epsilon }_{1},{\epsilon }_{2},{\epsilon }_{3}\right)$ is required because under zero-mean condition also, fourth- and second-order moments are needed to compute ${K}_{4}\left({\epsilon }_{1},{\epsilon }_{2},{\epsilon }_{3}\right)$ and it is represented as

14 $${K}_{4}\left({\epsilon }_{1},{\epsilon }_{2},{\epsilon }_{3}\right)={n}_{4}\left({\epsilon }_{1},{\epsilon }_{2},{\epsilon }_{3}\right)-{n}_{2}\left({\epsilon }_{1}\right).{n}_{2}\left({\epsilon }_{3}-{\epsilon }_{2}\right)-{n}_{2}\left({\epsilon }_{2}\right).{n}_{2}\left({\epsilon }_{3}-{\epsilon }_{1}\right)-{n}_{2}\left({\epsilon }_{3}\right).{n}_{2}\left({\epsilon }_{2}-{\epsilon }_{1}\right)-{n}_{1}\left[{n}_{3}\left({\epsilon }_{2}-{\epsilon }_{1},{\epsilon }_{3}-{\epsilon }_{2}\right)+{n}_{3}\left({\epsilon }_{2},{\epsilon }_{3}\right)+{n}_{3}\left({\epsilon }_{3},{\epsilon }_{1}\right)+{n}_{3}\left({\epsilon }_{1},{\epsilon }_{2}\right)\right]-{({n}_{1})}^{2}\left[{n}_{2}\left({\epsilon }_{1}\right)+{n}_{2}\left({\epsilon }_{2}\right)+{n}_{2}\left({\epsilon }_{3}\right)-{ n}_{2}\left({\epsilon }_{3}-{\epsilon }_{1}\right)+{n}_{2}\left({\epsilon }_{3}-{\epsilon }_{2}\right)+{n}_{2}\left({\epsilon }_{2}-{\epsilon }_{1}\right)\right]-6{({n}_{1})}^{4}$$

where ${n}_{4}\left({\epsilon }_{1},{\epsilon }_{2},{\epsilon }_{3}\right)$ is the fourth-order moment and if signal is having zero-mean, then,

15 $${K}_{4}\left({\epsilon }_{1},{\epsilon }_{2},{\epsilon }_{3}\right)={n}_{4}\left({\epsilon }_{1},{\epsilon }_{2},{\epsilon }_{3}\right)-{n}_{2}\left({\epsilon }_{1}\right).{n}_{2}\left({\epsilon }_{3}-{\epsilon }_{2}\right)-{n}_{2}\left({\epsilon }_{2}\right).{n}_{2}\left({\epsilon }_{3}-{\epsilon }_{1}\right)-{n}_{2}\left({\epsilon }_{3}\right).{n}_{2}\left({\epsilon }_{2}-{\epsilon }_{1}\right)$$

Fourth-order cumulant describes about the kurtosis of the signal. If these cumulants are considered in frequency domain then it can be obtained by taking the Fourier transform of these cumulants. Fourier transform of third-order cumulant is given as

16 $$S\left({\varphi }_{1},{\varphi }_{2}\right)=Z\left({\varphi }_{1}\right)Z\left({\varphi }_{2}\right){Z}^{*}\left({\varphi }_{1}+{\varphi }_{2}\right)=\sum_{{u}_{1}=-\infty }^{\infty }\sum_{{u}_{2}=-\infty }^{\infty }{K}_{3}\left({\epsilon }_{1},{\epsilon }_{2}\right).{e}^{-j\pi ({\varphi }_{1}{u}_{1}+{\varphi }_{2}{u}_{2})}$$

where $S\left({\varphi }_{1},{\varphi }_{2}\right)$ is the bispectrum of z(m), ${K}_{3}\left({\epsilon }_{1},{\epsilon }_{2}\right)$ is the third-order cumulant and $Z\left(\varphi \right)$ is the Fourier transform of x(n).

Similarly, for the fourth-order cumulant, its Fourier transform can be defined as Trispectrum and is given as

17 $$Q\left({\varphi }_{1},{\varphi }_{2},{\varphi }_{3}\right)=Z\left({\varphi }_{1}\right)Z\left({\varphi }_{2}\right)Z\left({\varphi }_{3}\right){Z}^{*}\left({\varphi }_{1}+{\varphi }_{2}+{\varphi }_{3}\right)=\sum_{{u}_{3}=-\infty }^{\infty }\sum_{{u}_{2}=-\infty }^{\infty }\sum_{{u}_{1}=-\infty }^{\infty }{K}_{4}\left({\epsilon }_{1},{\epsilon }_{2},{\epsilon }_{3}\right).{e}^{-j\pi ({\varphi }_{1}{u}_{1}+{\varphi }_{2}{u}_{2}+{\varphi }_{3}{u}_{3})}$$

where $Q\left({\varphi }_{1},{\varphi }_{2},{\varphi }_{3}\right)$ represents the Trispectrum of *z*(*m*) and ${K}_{4}\left({\epsilon }_{1},{\epsilon }_{2},{\epsilon }_{3}\right)$ as the fourth-order cumulant. Generalization of the cumulant parameters, with respect to the zero maximum lag to be computed, is shown in the Table 3.

**Table 3:** Generalization of the cumulant parameters.

Cumulants	Sample1	Sample2	Sample3
${K}_{3}\left({\epsilon }_{1},{\epsilon }_{2}\right)$	−0.37	0.21	1.98
${K}_{4}\left({\epsilon }_{1},{\epsilon }_{2},{\epsilon }_{3}\right)$	3.01	8.81	2.60
$S\left({\varphi }_{1},{\varphi }_{2}\right)$	0.94	1.33	1.45
$Q\left({\varphi }_{1},{\varphi }_{2},{\varphi }_{3}\right)$	2.08	1.64	0.72

Cumulants have been never used in the classification of ECG signals. In the approach utilized by V Sharmila et al*.*, 3rd-order cumulant was utilized there to obtain the symmetry in the signal which is further utilized for AR modelling which helps in enhancing the ECG signal^70^. This feature of any signal i.e. symmetry can be used for classification. This feature of the non-stationary signals can be useful in classifying the non-stationary beats in ECG signals and hence, exploited here to obtain the features from the ECG signals. Then, for the classification purpose, advanced version of neural network has been used and is explained in the next sub-section.

### Kernel extreme learning machine

Kernel extreme learning machine (KELM) is an extension of extreme learning machine (ELM). ELM was introduced by G.B. Huang in the year 2006^71^. It is a non-linear mapping process and was modelled for single feedforward hidden layer neural network^72^. Different from traditional neural network, ELM is a non-iterative approach and targets to minimize the training error and output weight’s norm. ELM has gained attention among active research topic since last one decade^66,67,73–76^. This is because of fusion of multiclass and binary classification, having ability to perform regression and classification, easy implementation and higher recognition rate.

An ELM model is defined for *r*-dimensional input vector having *w* number of training samples as $Y=\{\left({y}_{w},{\tau }_{w}\right)|w=1, \mathrm{2,3},4,\dots ,W\}$. As ${y}_{w}$ is *r*-dimensional, hence, input vector ${y}_{w}=[{y}_{w1},{y}_{w2},\dots ,{y}_{wr}]$ and the corresponding target class (*c* number of classes), ${\tau }_{w}=[{\tau }_{w1},{\tau }_{w2},\dots ,{\tau }_{wc}]$. Therefore, ELM model for *P* number of neurons in the hidden layer is

18 $${\mathrm{\rm Z}}_{ELM}\left(y\right)=\sum_{p=1}^{P}{\mu }_{p}.{\kappa }_{p}({\omega }_{p},{\beta }_{p},y)=\kappa \left(y\right).\mu $$

where ${\mu }_{p}={[{\mu }_{p1},{\mu }_{p2},{\mu }_{p3},\dots ,{\mu }_{pc}]}^{T}$ and ${\kappa }_{p}\left({\omega }_{p},{\beta }_{p},y\right)={\omega }_{p}y+{\beta }_{q}$. ${\mu }_{p}$ tells about the weight at the hidden node ${\kappa }_{p}$ output. $P, {\omega }_{p}$ and ${\beta }_{p}$ represent the number of neuron in the hidden layer, weight vector on the p^th^ neuron of that hidden layer and bias on the p^th^ neuron of the hidden layer respectively. The Eq. (18) can be re-written in the matrix form as:

19 $${\rm K}.\mu =\Gamma $$

where, $${\rm K}=\left[\begin{array}{ccc}{\mu }_{1}({\omega }_{1},{\beta }_{1},{y}_{1})& \cdots & {\mu }_{P}({\omega }_{P},{\beta }_{P},{y}_{1})\\ \vdots & \ddots & \vdots \\ {\mu }_{1}({\omega }_{1},{\beta }_{1},{y}_{W})& \cdots & {\mu }_{P}({\omega }_{P},{\beta }_{P},{y}_{W})\end{array}\right]$$, $$\mu =\left[\begin{array}{c}{\mu }_{1}^{T}\\ \vdots \\ {\mu }_{P}^{T}\end{array}\right]$$, $$\Gamma =\left[\begin{array}{ccc}{\tau }_{11}& \cdots & {\tau }_{1c}\\ \vdots & \ddots & \vdots \\ {\tau }_{W1}& \cdots & {\tau }_{Wc}\end{array}\right]$$

Thus, the output weight $\mu$ on the hidden node is given as the pseudo inverse of ${\rm K}$ and it is represented as

20 $$\mu ={{\rm K}^{T}\left(\frac{I}{{\mathbb{C}}_{\mathcal{R}}}+{\rm K}{\rm K}^{T}\right)}^{-1}\Gamma $$

This gave a model named as ELM model that can be shown as

21 $${\mathcal{M}}_{ELM}(y)={\kappa (y){\rm K}^{T}\left(\frac{I}{{\mathbb{C}}_{\mathcal{R}}}+{\rm K}{\rm K}^{T}\right)}^{-1}\Gamma $$

Here, ${\mathbb{C}}_{\mathcal{R}}$ is the regularization coefficient which is a constant and the value of this constant is required to be selected properly for the generalized performance of the model. ELM model has various advantages of lower computational complexity as the method is non-iterative, minimum error is achieved with the help of proper training. The problem of local minima and over-fitting is present in ELM. These problems are overcome by using kernel matrix with ELM, introduced in 2016 based on Mercer’s condition^74,77^, as

22 $${\chi }_{j,k}=\psi \left({y}_{j},{y}_{k}\right), j,k=\mathrm{1,2},3,\dots , W$$

Now, modifying the ELM model as represented using Eq. (21), gives,

23 $${\mathcal{M}}_{KELM}(y)={\kappa (y){\rm K}^{T}\left(\frac{I}{{\mathbb{C}}_{\mathcal{R}}}+\chi \right)}^{-1}\Gamma $$

and

24 $$\kappa \left(y\right){\rm K}^{T}=\left[\begin{array}{c}\psi \left(y,{y}_{1}\right)\\ \vdots \\ \psi \left(y,{y}_{W}\right)\end{array}\right]$$

$\kappa (y)$ is that hidden node output which maps the input data to the hidden layer feature space. If there are two samples, say ${\gamma }^{th}$ and ${\delta }^{th}$ input samples, then the kernel function can be stated as

25 $$\psi \left({y}_{\gamma },{y}_{\delta }\right)=\kappa ({y}_{\gamma }){\kappa ({y}_{\delta })}^{T}$$

There are various kernel functions which can be used in the kernel based ELM. They are polynomial kernel, Laplacian kernel, sigmoid kernel, wavelet kernel, and RBF kernel. Equations for these kernel functions are shown in Table 4.

**Table 4:** Kernel functions with their equations.

Equation	
Polynomial	$\psi \left(x,y\right)={\left({x}^{T}y+1\right)}^{n}$
Laplacian	$\psi \left(x,y\right)={e}^{\left(-\Vert x-y\Vert /\sigma \right)}$
Sigmoid	$\psi \left(x,y\right)=\mathrm{tanh}(\beta {x}^{T}y+c)$
Wavelet	$\psi \left(\overrightarrow{x},\overrightarrow{y}\right)=\prod_{\varsigma =1}^{r}\left(\phi \left(\frac{\overrightarrow{{x}_{\varsigma }}-\overrightarrow{{y}_{\varsigma }}}{\nu }\right)\right)$
RBF	$\psi \left(x,y\right)= {e}^{\left(-{{\rm P}_{\rm K}\Vert x-y\Vert }^{2}\right)}$

**Figure 5:**
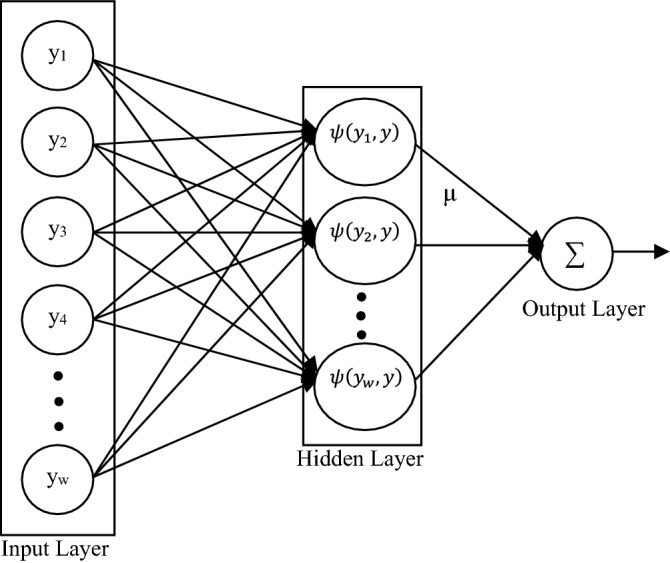
Architecture of KELM.

Any of these kernel functions can be utilized with KELM depending upon the requirement and hence, the kernel based ELM model is defined as the kernel extreme learning machine (KELM). Its architecture is shown in Fig. 5.

KELM can be utilized for both binary as well as multiclass classification^78^. Here, it is used in multiclass classification of ECG signals. This classification of ECG signals is optimized for minimum percentage error rate using an optimization algorithm which is explained in the next sub-section. In KELM, regularization coefficient and kernel parameter are the two variables whose optimized values affect the recognition of ECG signals.

### Optimization algorithm

Optimization algorithm helps in selecting such values of the parameters or variables at which percentage error rate can be minimized in order to achieve good rate of ECG signals classification. This is performed using genetic algorithm (GA) here. GA was introduced by Holland and Goldberg using the concepts of genetics and Darwin’s theory^79^.

**Figure 6:**
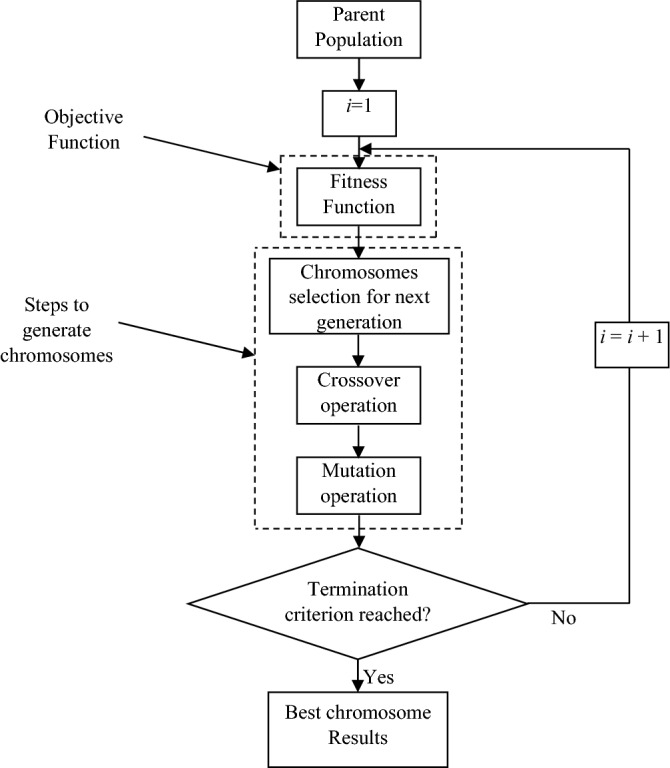
Basic structure of GA.

In GA, fitness function is used to check for the best solution. It is a function which takes the solution (chromosome) as input and provides solution as output. Various combinations of the parameters are formed and tested for the solutions to the problem. Combinations of these parameters are selected using three basic steps of GA. These are as follows: parent selection, crossover and mutation. A basic structure representing the algorithm of GA is shown in Fig. 6.

#### Selection

It is a process in GA with which initial variables are selected as parent variables and they are mated and recombined to produce their off-spring or child. It is very important step as a good selection of the parent variable helps in generating better off-springs for the better solution. Selection can be done in various ways: fitness proportionate selection, tournament selection, stochastic uniform sampling, roulette wheel selection, random selection and rank selection. An appropriate selection is must to achieve better and fit solutions. Improper selection will lead to premature convergence to a suboptimal solution which is because of getting stuck in local minima. This problem may also arise because of the small size of the population. Thus, it is necessary to have a good selection of initial variables so that better off-springs can be generated and lead to achieve better results.

#### Crossover

It is the next step to selection in which one of the several crossover operators is utilized on the selected parents and off-springs are produced using the genetic properties of the parents. These operators include uniform crossover, partially mapped crossover, Davis’ Order crossover, shuffle crossover, whole arithmetic recombination, ring crossover, order based crossover, one point crossover, and multi point crossover. Various combinations of parent chromosomes are performed to obtain the child chromosomes using these operators.

#### Mutation

It is defined as a fine adjustment in the child chromosome to obtain a whole new chromosome. It is performed to have diversity in the genetic population so that search space can be explored widely. It is an essential step for the convergence of GA. It also uses some operators for their function that commonly includes swap mutation, inversion mutation, scramble mutation, random resetting, and bit flip mutation. These operators are utilized according to the requirement in the problem that need to be solved.

In GA, population is initiated either randomly or with some other heuristics and parent chromosomes are selected for mating. Value of the fitness function (or objective function) is computed. Now, crossover and mutation operators are applied on the parent chromosomes to produce child chromosomes. Fitness function value is computed again for these child chromosomes. Both the values are compared and chromosomes with which best solution is obtained will help in generating the chromosomes for next generation. This step repeats until termination criterion is reached.

Termination criterion is very important in GA to end a GA running process. Some conditions that can be utilized to stop a GA run are, when number of iterations (or generations) reached to maximum, when population size becomes equal to the chromosomes validated, or when best fitness function value becomes equal to the mean of the fitness function values of all the iterations.

### Databases used

The proposed approach is experimented over various kinds of ECG databases. These include MLII, UCI Repository arrhythmia and PTBDB database. All these databases are briefly explained as follows:

#### MLII database

MLII database of ECG is obtained from MIT-BIH arrhythmia database of the PhysioNet services^80^. At PhysioNet, 48 records are present but all the records are not considered because record no. 102 and 104 do not contain the MLII lead output and record no. 232 is the Sinus bradycardia rhythm in the entire signal. With MIT-BIH arrhythmia database of 45 records, all the signals are obtained from one lead i.e. MLII and hence, named as MLII ECG database. It is having 17 classes with 1000 fragments in each signal. Normal ECG, pacemaker rhythm and 15 other types of cardiac disorders are the classes in MLII ECG database. Names of each class are given in Table 1 with respective number of samples in each class. All the samples of the ECG signals contain 3600 attributes recorded at 200 adu/mV gain and 360 Hz sampling frequency with 10 s non-overlapping fragments.

#### UCI repository arrhythmia database

UCI repository arrhythmia database is one of another ECG database that is experimented to check the efficiency of the proposed approach. This database consists of 452 samples distributed in 13 classes. Class 1 is having the normal ECG data, classes 2 to 15 shows different cardiac disorders and Class 16 contains the ECGs which are not classified in any of the categories. Each sample contains 280 attributes. Out of all these attributes, first four attributes represents general details about the sample viz. age, sex, height and weight, while rest 275 attributes are the parametric details of the ECG signal including duration of QRS complex, duration between onset P and Q waves, between Q and offset T waves, duration between two consecutive P waves etc. and 280^th^ attribute tells about the class to which that sample belongs^60^. Out of all attributes, 206 are linear valued attributes and 73 as the nominal ones. All of these values are taken in milliseconds duration that represents average values. These parametric values are taken from a 12-lead recording of ECG^81^. Name of these arrhythmia classes with their respective number of samples is given in Table 5. In this ECG database, 11th to 15th attributes in each class, contains the missing values^82^. These missing values are filled with some values, the process of that is given and explained in the next section.

**Table 5:** Comparison of number of samples in the classes in balanced UCI repository arrhythmia corrected database with number of samples in the classes in unbalanced UCI repository arrhythmia corrected database.

S.No	Class	Unbalanced database	ROST	IRST	RUST
1	Normal	245	245	35	2
2	Ischemic changes (coronary artery disease)	44	245	35	2
3	Old anterior myocardial infarction	15	245	35	2
4	Old inferior myocardial infarction	15	245	35	2
5	Sinus tachycardia	13	245	35	2
6	Sinus bradycardia	25	245	35	2
7	Ventricular premature contraction (PVC)	3	245	35	2
8	Supraventricular premature contraction	2	245	35	2
9	Left bundle branch block	9	245	35	2
10	Right bundle branch block	50	245	35	2
11	Left ventricule hypertrophy	4	245	35	2
12	Atrial fibrillation or flutter	5	245	35	2
13	Others	22	245	35	2

#### PTBDB ECG database

The Physikalisch-Technische Bundesanstalt Database or PTBDB is also taken from the PhysioNet’s bank. This ECG database is categorized into two classes containing the signals representing the shapes of ECG heartbeats. Two classes consist of normal ECG class having 4046 numbers of samples and abnormal ECG class with 10,506 numbers of samples. Abnormal samples are the cases affected with myocardial infarction and other different arrhythmias. Hence, there are total 14,552 numbers of samples present in PTBDB database. All the samples of the signals are already preprocessed and each sample is segmented and sampled at a frequency of 125 Hz to represent an individual’s heartbeat. Thus, all the signals of PTBDB ECG database are cropped, down-sampled and padded with zeros to make the dimension of each signal equal to 188^83^*.*

## Proposed method

**Figure 7:**
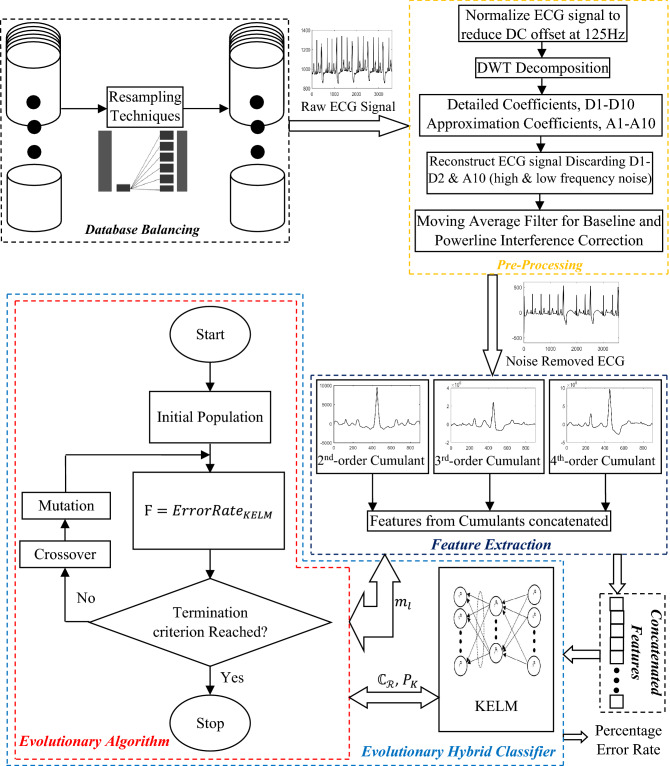
Detailed block diagram of the proposed approach.

The proposed approach introduces a novel and robust approach of ECG signals classification. It is based on feature vectors obtained with the help of cumulants. 2nd-, 3rd- and 4th-order cumulants are utilized as the statistical approach of feature extraction. As it is already stated, 2nd-order cumulant is helpful in computing the autocorrelation of the signal, similarly, 3rd- and 4th-order cumulants for skewness and kurtosis of the signal respectively. These are very useful properties of non-stationary signals such as ECG because any small variation in the health of a person can be seen as variation in its ECG. Such variations can be computed statistically and helpful in recognizing different types of ECG signals. For the faster speed of the proposed approach, non-iterative method is used for classification. This non-iterative method is hybridized with optimization algorithm and hence, forms an evolutionary hybrid classifier. A block diagram of the proposed approach is shown in Fig. 7.

**Figure 8:**
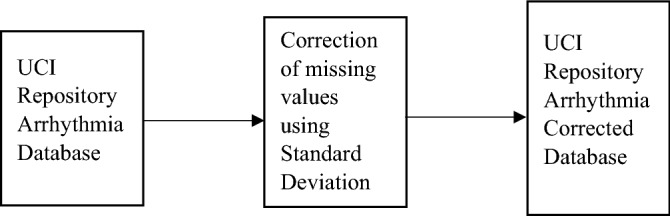
Missing attributes corrected in UCI repository arrhythmia database.

Here, three databases are utilized for checking the robustness of the proposed approach. These are explained in “Databases used”. MLII and PTBDB databases are the proper ECG signals having 1000 fragments in MLII and PTBDB cropped and down-sampled to 188 dimension sizes. UCI repository database contains ECG parameters as stated above in “UCI repository arrhythmia database”. In this database, as there are some missing values in 11th to 15th attributes, therefore, to maintain the relevance and reliability of the arrhythmia database, it is a prior need to fill these missing values by pre-processing the database. In some of the earlier researches, these missing values are tackled by directly removing the rows containing these values. Researchers also removed the 13th class which have uncertainty about the class containing unrecognized data. We have utilized this data as the separate class as 13th class. The missing values are dealt with a pre-processing step as it is shown in Fig. 8. As shown in figure, the missing values in 11th to 15th attributes of UCI repository arrhythmia database are replaced by the standard deviation of all remaining attributes of the respective class. The database containing these corrected values in the missing attributes is termed as the UCI repository arrhythmia corrected database. MLII & PTBDB ECG database do not have any such attributes or missing values, therefore, no correction is required in these two databases.

Now, there are three databases of three different types. One is the complete ECG signal having 1000 fragments as in MLII, second is the ECG data down-sampled to 188 size of dimension and third one having parameter values of ECG signals. All these three databases are unbalanced having one class as the majority in number compared to other classes. Therefore, all three databases are balanced using resampling techniques. ROST and IRST are utilized as resampling techniques here. RUST is avoided because it removes the samples from the classes and reduces the database to very small size. Table 6 is showing the number of samples in the classes, before and after applying resampling techniques, of various databases used during experimentation. After balancing the databases, noise removal is performed on the signals of ECG databases. It is performed utilizing DWT. ECG signal’s biology and shape helps in selecting the mother wavelet and the level of decomposition required^84^. As the Daubechies (db6) wavelet resembles the most with ECG signal, hence, it is used with decomposition level 10. There are many disturbances present in a raw ECG signals. These disturbances are present due to motion artifacts, power line interference and skin electrode contact^85^. This is performed normalizing the ECG signal so that DC offset (125 Hz) can be reduced^86^ and variance of the amplitude can be eliminated.

**Table 6:** Total number of samples before and after applying resampling techniques in various Databases used for experimentation.

Technique →	Imbalance ratio	Before	ROST	IRST	RUST
Databases ↓					
MLII	283:10	1000	4811	1003	170
UCI	245:2	452	3185	455	26
PTBDB	309:119	14,552	21,010	14,552	8092

After that signal is denoised using Daubechies wavelet of vanishing moments six and ten level of decomposition. WT decomposes the ECG signal into detailed and approximation coefficients as shown in Fig. 4. Then, high frequency noise is removed from the signal by removing the detailed coefficients D1-D2 and low frequency noise is removed by eliminating the low frequency coefficient A10. It is done with the help of automatic soft computing technique. ECG signal is regenerated by combining the rest of the coefficients. One more noise is still present in the signal i.e. Baseline wander noise at the range of 0.15–0.8 Hz. This noise is due to the electrode impedance and respiration in the human body^86^. It is removed with the help of moving average filter and signal has been smoothed. This step of pre-processing in the proposed approach is shown in Fig. 7.

These smoothened ECG data is then utilized for extracting the features. These features are statistical measures in terms of cumulants. 2nd-, 3rd- and 4th-order cumulants are computed of the smoothened ECG data means the features are extracted from noise removed ECG signals. If any noise or disturbance is still present then these features will be helpful in such conditions. This is because results the 3rd- and 4th-order cumulants are insensitive towards noise. Pre-processing step of noise removal with these higher order cumulants makes the proposed method more robust towards the ECG signals used. Also, 2nd-, 3rd- and 4th-order cumulants are applied in this proposed method because 2nd-order cumulant tells about the autocorrelation of the signal, 3rd-order cumulant tells about the skewness and 4th-order cumulant tells about the kurtosis of the ECG signal. 2nd-order cumulant or autocorrelation does not contain any information about phase^70^. With this advantage of minimum phase, 2nd-order cumulants are greatly helpful in identifying the non-linear signals like ECG signals. There are some types of phase coupling associated with nonlinear signals that are not correctly identified with the help of 2nd-order cumulants. In such conditions, higher order cumulants are useful. 3rd-order cumulant or skewness is a measure of asymmetry of any distribution about its mean^70^. It can have positive and zero values only. Positive value of skewness tells that the tail of the ECG signal is longer and thinner towards right side as compared to the left side. Zero value of skewness depicts the case of symmetric signal about the mean. It is also true in asymmetric signals in which asymmetry obeys, one tail being short and thin, and the other being long and thick. In the ECG waveforms, some kind of asymmetry is observed among four types of ECG datasets used. 4th-order cumulant or kurtosis of the signal which is a measure of the peakedness in the distributions and peakedness of any ECG waveform is defined by width of their peaks^70^. Higher kurtosis means more of the variance which is the result of infrequent extreme deviations and their Fourier transforms gives Bispectrum and Trispectrum for the signals, respectively, which can also be used as features for the signals. Hence, 2nd-, 3rd- and 4th-order cumulants are used for feature extraction to achieve better accuracy and classification results. In the following equation, size of the feature vector (*N*_*K*_) obtained using cumulant is represented^87^:

26 $${N}_{K}=2*{m}_{l}+1$$

where, ${m}_{l}$ is the maximum number of lags of the cumulant that need to be used. The classification is performed with the help of evolutionary hybrid classifier. It is hybrid of optimization algorithm, GA and non-iterative algorithm i.e. KELM. Algorithm for the evolutionary hybrid classifier is also given.

In the evolutionary hybrid algorithm, parameters of KELM are optimized using GA. For this, population of the parameters (${m}_{l}$, ${\mathbb{C}}_{\mathcal{R}}$, ${\rm P}_{\rm K}$) are initialized, termination criterion is set with lower and upper limits of the ${\mathbb{C}}_{\mathcal{R}}$ and ${\rm P}_{\rm K}$. Fitness function for the algorithm is error rate computed using the classifier KELM. It is written below in the form of equation:

27 $${\rm F}={ErrorRate}_{KELM}$$

*ErrorRate*_*KELM*_ is defined as the total number of incorrect predictions divided by the total number of data samples in the database. Here, in Using confusion matrix, it is defined as

$$ ErrorRate_{KELM} \, = \,\left( {{\text{FP}}\, + \,{\text{FN}}} \right)/\left( {{\text{TP}}\, + \,{\text{FN}}\, + \,{\text{FP}}\, + \,{\text{TN}}} \right) $$

Confusion matrix:

**Table:** 

TP	FN
FP	TN

where, TP is correct positive prediction, FN is incorrect positive prediction, FP is correct negative prediction, TN is incorrect negative prediction

**Figure 9:**
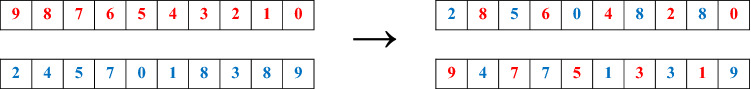
Crossover operation in evolutionary hybrid classifier.


The fitness function value is computed for the initial population and the best fit from that is obtained. The best fit is the values of parameters (${m}_{l}$, ${\mathbb{C}}_{\mathcal{R}}$, ${\rm P}_{\rm K}$) for the minimum Ϝ. Then these best fit chromosomes will become the parent for the next generation. Next generation population is obtained using crossover and mutation operations. Uniform crossover operator is applied in which each gene is treated separately without dividing the chromosomes into the segments. A representation is shown in Fig. 9 for uniform crossover.

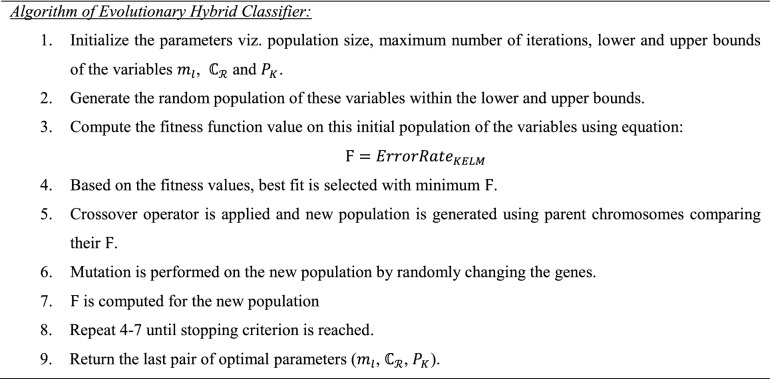


**Figure 10:**
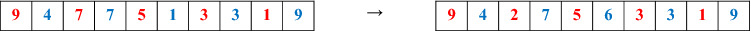
Mutation operation in evolutionary hybrid classifier.

After that, the resultant chromosomes are applied with mutation operator i.e. random resetting operator here. In this, one or more genes are selected and there values are replaced with other random values within the given range. It is shown, in Fig. 10, as follows:

Again, the fitness function is computed for the new generation and best fit is computed using Ϝ. Same process is repeated until some termination criterion is reached or the best fit (or minimum Ϝ) becomes equal to the mean value of the Ϝ obtained from the last generated population when the run is terminated. Hence, the optimized values of the parameters ${m}_{l}$, ${\mathbb{C}}_{\mathcal{R}}$ and ${\rm P}_{\rm K}$ are obtained with corresponding percentage error rate (best fit or minimum Ϝ).

## Experimental results and analysis

The proposed approach of ECG signals classification undergoes various steps in experimentation. For achieving better results in identifying the signal, pre-processing and feature extraction are performed which provides precision to the proposed approach. Experimental analysis performed on the proposed approach is explained as follows:

As it is already mentioned in “Databases used”, three different types of ECG signals are utilized for the analysis of the proposed approach. All these databases are facing a problem of large difference in number of samples in majority and minority classes. Imbalance ratio for each ECG database, used during experimentation, is given in the Table 6. Three databases MLII, UCI Repository Arrhythmia Corrected and PTBDB database have imbalance ratio of 283:10, 245:2 and 309:119 respectively. Imbalanced databases make the classification biased towards the majority class. Therefore, resampling techniques are applied on these databases to balance the number of samples in majority class with minority class. Comparison of number of samples in the classes in balanced database with number of samples in the classes in imbalanced database is represented in Tables 1 and 2.

It can be seen from the tables (Tables 1 and 2) that RUST is not an appropriate resampling technique as it reduces the number of samples leading to loss of data which makes the system unreliable. ROST and IRST techniques do not suffer loss of data as they add samples to the classes. Therefore, ROST and IRST techniques are utilized in the proposed approach for balancing the number of samples in the classes of databases. ROST has a limitation of over-fitting because of addition of large number of samples. This problem is overcome by using the evolutionary hybrid classifier. After data balancing, pre-processing is performed with the help of DWT.

**Figure 11:**
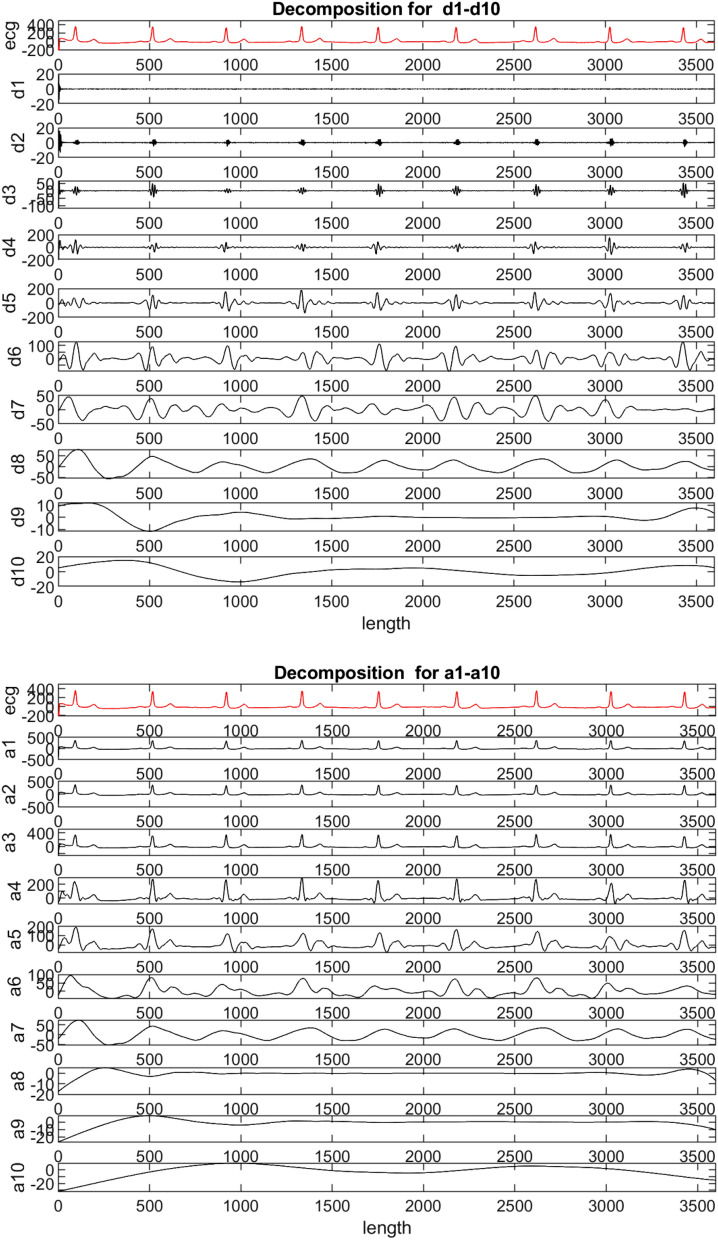
Decomposition for d1–d10 and a1–a10 of ECG Signal using db6 level 10 DWT.

ECG signals are affected with many noises, as already explained in previous section (“Proposed method”). Therefore, noise is removed from these signals using db6 level 10 WT. DWT decomposition for an ECG signal is shown in Fig. 11. Detailed (D1–D10) and approximation (A1–A10) coefficients are represented in the figure. It is the segregation of a signal into various frequency bands so that time–frequency information of the signal can be extracted. As it can be seen in Fig. 11, A10 contains the lowest frequency band of the ECG signal and D1 contains the highest frequency band. These are the noises in the signal and hence, removed by filtering these particular bands. A10 is targeted for low frequency noise removal.

**Figure 12:**
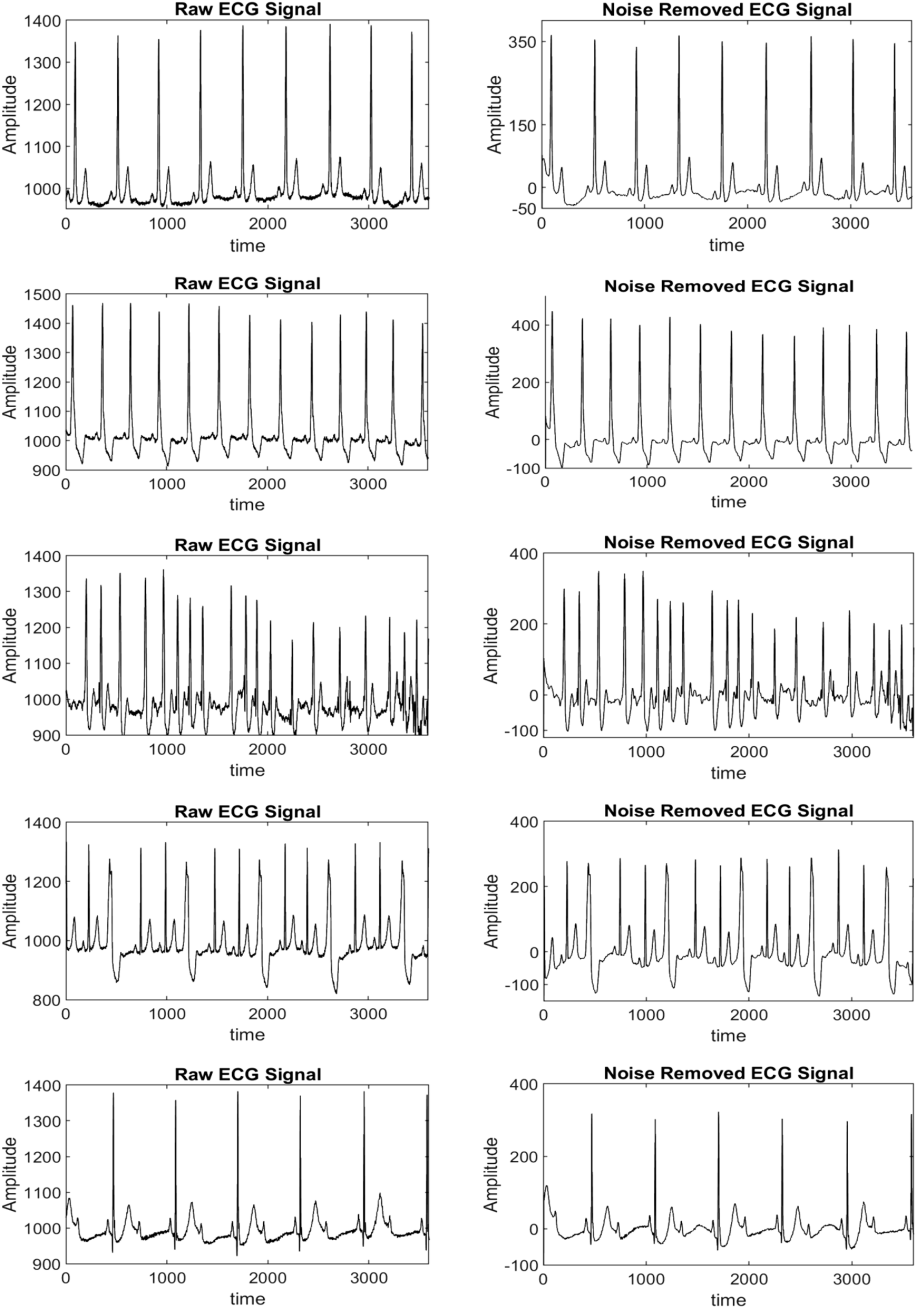
Raw ECG signals and noise removed ECG signals.

For high frequency noise removal, D1 and D2 are removed. Baseline correction and powerline interference noises are also performed at 0.15–0.8 Hz and 60 Hz respectively. The ECG signal after removing all these noises is reconstructed as noise removed ECG signals. It is shown in Fig. 12. In this figure, five samples of original ECG signals are shown and correspondingly, noise removed ECG signals are represented. After filtering the noise, next step is to compute features from this noise free ECG signal. Therefore, 2nd-, 3rd- and 4th-order cumulants comes into action as feature extractors.

**Figure 13:**
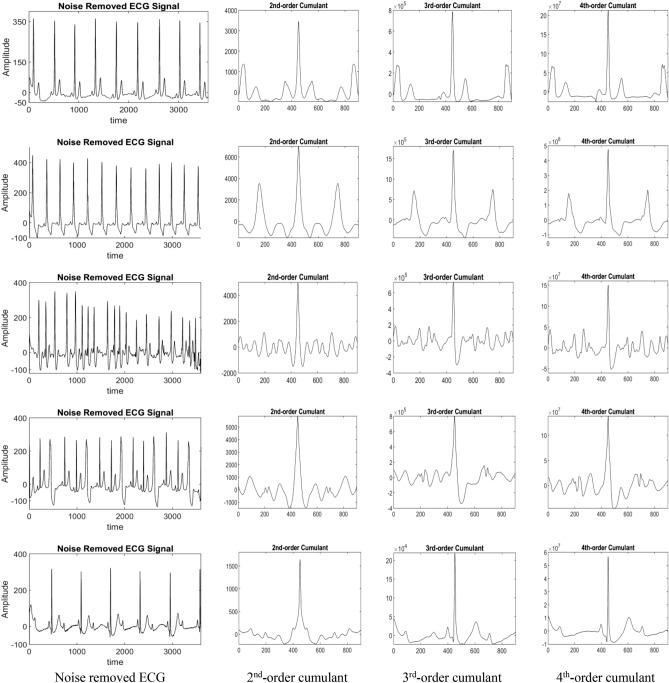
Noise removed ECG and corresponding 2nd-, 3rd- and 4th-order cumulants.

2nd-, 3rd- and 4th-order cumulants are used here for obtaining the statistical features from the ECG signals. These are shown as samples in Fig. 13. Five sample of noised free ECG signals are shown with their corresponding 2nd-, 3rd- and 4th-order cumulants. As it can be seen from the figure, there are variations in the curves obtained by applying these multi-cumulants. These variations are because of variations in the ECG signal due to various types of arrhythmia problems. These arrhythmia problems creates disturbances in the ECG of a subject which are reflected in 2nd-, 3rd- and 4th-order cumulants. It also represents that using a single type of cumulant is not sufficient as a feature vector as it is unable to differentiate between different types of ECG signals. 3rd- and 4th-order cumulants work better in non-linear signals like ECG. Therefore, concatenating 2nd-, 3rd- and 4th-order cumulants will give the feature vector of size, computed by multiplying Eq. (26), by 3 (for concatenation of three cumulant’s features) for various ECG signals in the databases. These feature vectors are then utilised to classify the ECG signals with the help of an evolutionary hybrid classifier. This classifier uses KELM which is a non-iterative algorithm and overcomes the problem of overfitting that is generated because of ROST. Kernel function selected for the experimentation is RBF kernel. Its equation is shown in Table 4. Evolutionary algorithm helps in optimization of the parameters ${m}_{l}$, ${\mathbb{C}}_{\mathcal{R}}$ and ${\rm P}_{\rm K}$ with the help of GA. Now, based on this proposed approach, classification of different types of ECG signals is represented according to the databases used for the experimentation.

### MLII ECG database

After applying balancing, pre-processing and feature extraction to the MLII ECG Database, classification of the ECG signals is performed based on the feature vectors obtained with vector size (2703 units) using Eq. (26). Size of the original signal (size 3600 units) is reduced to 2703 units; hence, dimension reduction is also obtained with the proposed approach. As shown in Table 1, after balancing using IRST technique each class of the MLII database have 59 samples in each class owing to a total of 1003 samples. These are divided into Train-Test ratio. Combination of ratios taken for this database are as follows: 20–80, 30–70, 40–60, 50–50, 60–40, 70–30, 90–10 and 95–05. Taking one by one each of these ratios, KELM is trained, following which testing is performed on the test samples. Values of KELM parameters (${\mathbb{C}}_{\mathcal{R}}$, ${\rm P}_{\rm K}$) and feature vector parameter, ${m}_{l}$ are obtained by optimizing KELM using GA. All the results obtained for this database (for IRST based balancing of database) are shown in Table 7. Values of ${m}_{l}$, ${\mathbb{C}}_{\mathcal{R}}$ and ${\rm P}_{\rm K}$ are also shown in the table. It can be seen in the table as well that as the training data is increasing, percentage error rate is getting reduced and best value is achieved with 1.96% error rate indication 1 ECG signal misclassification out of 50 ECG signals. If taking 70–30 Train Test ratio case, 3.59% error rate is obtained. It means only 11 ECG signals are misclassified out of 301 ECG signals.

**Table 7:** Percentage error rate with values of ${m}_{l}$, ${\mathbb{C}}_{\mathcal{R}}$ and ${\rm P}_{\rm K}$ obtained using proposed approach on MLII ECG database.

	Train-test	${m}_{l}$	${\mathbb{C}}_{\mathcal{R}}$	${\rm P}_{\rm K}$	Percentage error rate (%)
MLII ECG Database with IRST balancing	20–80	450	0.2104	23.645	11.14
	30–70	450	−2.984	5.634	9.33
	40–60	450	−44.584	83.677	6.55
	50–50	450	3.231	45.454	5.27
	60–40	450	4.364	35.442	3.68
	70–30	450	9.655	50.412	3.59
	90–10	450	−28.033	76.213	2.94
	95–05	450	−22.353	64.570	1.96
MLII ECG Database with ROST balancing	20–80	450	35.241	0.0567	11.43
	30–70	450	1.000	0.001	7.90
	40–60	450	0.222	0.0413	2.63
	50–50	450	14.622	0.001	0.00
	60–40	450	23.515	0.001	0.00
	70–30	450	51.232	0.001	0.00
	90–10	450	5.277	0.01	0.00
	95–05	450	1.595	0.1	0.00

Similarly, experiment is performed on MLII ECG Database when data balancing is performed using ROST. In that case, 283 ECG signals are present in each class with 4811 signals in total. Same sequence of Train-Test Ratio is taken here as well and results are computed by training of KELM and optimizing ${m}_{l}$, ${\mathbb{C}}_{\mathcal{R}}$ and ${\rm P}_{\rm K}$ using GA. Percentage error rate with their corresponding values of optimized ${m}_{l}$, ${\mathbb{C}}_{\mathcal{R}}$ and ${\rm P}_{\rm K}$ are shown in Table 7. Results obtained in this case are excellent as zero error rate is achieved on MLII ECG Database for this case. Zero error rate is achieved for 50–50 Train-Test ratio or when the training data is more than 50%. For 40–60 Train-Test ratio, 2.63% error rate is achieved. It means 16 ECG signals are misclassified out of 602 ECG signals.

### UCI repository arrhythmia database

The UCI repository arrhythmia database is different from MLII ECG database. As discussed in “Databases used”, UCI repository arrhythmia database is containing the parametric observations like P, Q, R, S, T peak values, their time durations, distance between various peaks etc. of the ECG signals. After performing same operations of the proposed approach on this database, two databases are selected in UCI repository arrhythmia corrected database i.e. balanced through IRST and ROST. In the first case, with IRST, 35 samples of ECG signals are there in each class of the database owing to 455 samples in total. The feature vector of each sample is 153 units (Eq. 26). Dimension reduction is achieved as 279 features of a sample are reduced to 153 features here. Train-Test ratio is again selected sequentially like 10–90, 20–80, 30–70, 40–60, 50–50, 60–40, 70–30, 80–20 and 90–10. In this also, KELM is trained with each training set and correspondingly testing is performed with remaining test set. Results are shown in terms of percentage error rate in Table 8. The values of the KELM parameters (${\mathbb{C}}_{\mathcal{R}}$, ${\rm P}_{\rm K}$) and the feature extraction parameter, ${m}_{l}$ are also obtained by optimizing GA to achieve minimum error rate. As with increasing training data, error rate reduces, therefore, 90–10 Train-Test ratio provides best result with 10.96% error rate in classifying the UCI repository arrhythmia corrected database when IRST is used for data balancing. It means 5 arrhythmias are misclassified out of 46. Similarly, for 70–30 Train-Test ratio, 16.92% error rate is achieved.

**Table 8:** Percentage error rate with values of ${m}_{l}$, ${\mathbb{C}}_{\mathcal{R}}$ and ${\rm P}_{\rm K}$ obtained using proposed approach on UCI repository arrhythmia corrected database.

	Train-test	${m}_{l}$	${\mathbb{C}}_{\mathcal{R}}$	${\rm P}_{\rm K}$	Percentage error rate (%)
UCI repository arrhythmia corrected database with IRST balancing	10–90	25	52.582	40.278	39.70
	20–80	25	15.560	3.732	30.77
	30–70	25	30.370	73.041	27.88
	40–60	25	16.852	4.578	20.15
	50–50	25	12.230	5.353	19.91
	60–40	25	8.285	8.253	18.68
	70–30	25	20.220	0.567	16.92
	80–20	25	−5.810	0.059	13.19
	90–10	25	−1.623	0.0225	10.26
UCI repository arrhythmia corrected database with ROST balancing	10–90	25	10.623	0.00008	8.25
	20–80	25	18.776	0.00008	0.12
	30–70	25	5.428	0.00008	0.00
	40–60	25	38.124	0.00008	0.00
	50–50	25	7.694	0.00008	0.00
	60–40	25	3.555	0.00008	0.00
	70–30	25	4.778	0.00008	0.00
	80–20	25	60.667	0.0001	0.00
	90–10	25	8.354	0.0001	0.00

While, in the second case, when ROST is utilized for resampling the database, results achieved are excellent. In this, 3185 samples are present in this database with 245 samples of each class. Zero error rate is achieved when training data 30% or more is used. It means with only 30% of training data, zero error rate is achieved on 70% test data. For 20–80 Train Test ratio, 0.12% error rate is obtained that means 3 arrhythmias are misclassified out of 2548 arrhythmias. Table 8 is representing the percentage error rate with their corresponding values of ${m}_{l}$, ${\mathbb{C}}_{\mathcal{R}}$ and ${\rm P}_{\rm K}$.

### PTBDB ECG database

PTBDB ECG Database is somewhat different from MLII and UCI databases as already discussed in detail in “Databases used”. It is cropped and down-sampled to 187 units of size. It means the size of a sample in this database is 187. This size further reduced by applying the proposed approach operations of pre-processing and feature extraction and the resultant size of the feature vector for each sample of PTBDB database is reduced to 153 units (Eq. 26). This database also undergoes with resampling techniques to maintain the imbalance ratio. After applying the resampling techniques, databases obtained from IRST and ROST are only utilized for further processing. Therefore, in IRST based balanced PTBDB database have 14,552 samples of ECG signal with 7276 samples in each class. In this database, there are only two classes i.e. normal and abnormal. Here also, Train-Test ratio is varied sequentially from 10–90 to 90–10. Results obtained on with this database are very good as 0.76% error rate is achieved with 90–10 Train-Test ratio. It means 11 ECGs are misclassified out of 1455. With Train-Test ratio 70–30, 0.73% error rate is achieved means 32 ECGs are misclassified out of 4366 ECGs. Results on PTBDB ECG Database are shown in Table 9. The values of the parameters ${m}_{l}$, ${\mathbb{C}}_{\mathcal{R}}$ and ${\rm P}_{\rm K}$ are also shown in the table with each Train-Test ratio for corresponding percentage error rate.

**Table 9:** Percentage error rate with values of ${m}_{l}$, ${\mathbb{C}}_{\mathcal{R}}$ and ${\rm P}_{\rm K}$ obtained using proposed approach on PTBDB ECG database.

	Train-test	${m}_{l}$	${\mathbb{C}}_{\mathcal{R}}$	${\rm P}_{\rm K}$	Percentage error rate (%)
PTBDB ECG database with IRST balancing	10–90	25	64.079	4.098	3.83
	20–80	25	28.729	4.296	2.59
	30–70	25	52.603	4.931	2.03
	40–60	25	95.160	6.369	1.35
	50–50	25	64.269	5.179	1.17
	60–40	25	71.480	4.190	0.96
	70–30	25	108.520	3.394	0.73
	80–20	25	9.428	3.476	0.72
	90–10	25	86.198	4.672	0.76
PTBDB ECG database with ROST balancing	10–90	25	188.257	4.854	3.14
	20–80	25	157.087	6.998	1.65
	30–70	25	67.544	5.023	1.06
	40–60	25	199.869	4.163	0.63
	50–50	25	189.643	5.130	0.57
	60–40	25	178.320	5.052	0.43
	70–30	25	92.776	4.767	0.35
	80–20	25	129.147	3.264	0.33
	90–10	25	190.023	3.286	0.43

Similar operation is performed with ROST based balanced PTBDB ECG Database. After processing through pre-processing step and features extraction, the database is fed to evolutionary hybrid classifier. In this case, 21,010 samples are present with 10,505 in each class. The database becomes quite large still excellent results are achieved on this database when it is experimented with proposed approach. Sequentially dividing the Train-Test ratio from 10–90 to 90–10, the database is divided into train and test set. The results are shown in Table 9. Best value of result achieved is with 90–10 Train-Test ratio i.e. 0.43% error rate. It means nine signals are misclassified from 2101 signals of PTBDB Database. Similarly, with 70–30 ratio, 0.35% error rate is achieved giving 22 misclassifications out of 6303. According to the size of this database, the results achieved are excellent with the proposed approach of evolutionary hybrid classifier.

### Comparison with other approaches

The results obtained on MLII, UCI and PTBDB ECG databases using proposed approach are compared with the existing state-of-art approaches. It is represented in Table 10. The performance measure selected for showing the ECG classification is percentage accuracy and it can be seen that excellent results have been achieved over these utilized databases.

**Table 10:** Comparison of the proposed approach with existing state-of-art approaches.

Approach	Year	Database	No. of classes	Train-test ratio	Accuracy (%)
Kernel difference weighted KNN^93^	2008	UCI	13	90–10	70.66
Modular neural network^81^	2011	UCI	13	90–10	82.22
Naïve Bayes^94^	2014	PTBDB	2	80–20	94.70
RBF SVM^95^	2015	PTBDB	2	90–10	96.00
KICA + LIBSVM^88^	2016	MLII	5	50–50	97.78
CNN^96^	2017	PTBDB	2	90–10	93.50
Third-order tensor based analysis ^50^	2017	PTBDB	2	80–20	95.30
Wrapper method^97^	2017	UCI	13	80–20	74.47
1-D CNN^65^	2018	MLII	17 15 13	70–30	91.33 92.51 95.20
PCAnet + SVM^89^	2018	MLII	5	90–10	97.77
CNN + LSTM^90^	2018	MLII	5	90–10	98.10
Evolutionary-neural system based on SVM^64^	2018	MLII	17 15 13	70–30	90.00 91.00 95.00
Deep neural network^98^	2018	PTBDB	2	90–10	95.90
Kernel extreme learning machine^67^	2018	UCI	13	90–10	78.26
Without feature extraction + CNN With feature extraction + CNN^57^	2019	PTBDB	2	90–10	94.03 98.37
GWMD-DE technique^59^	2019	UCI	13	50–50	96.00
Wavelet KELM^99^	2019	PTBDB	2	90–10	95.00
KELM with GA^66^	2020	UCI	13	90–10	86.67
WT-HMM mode ^91^	2020	MLII	5	90–10	99.80
DEA-ELM^100^	2020	PTBDB	2	90–10	97.50
Ensemble SVM^92^	2020	MLII	4	60–40	94.40
Ensemble SVM with GA-SVM features selection^101^	2020	UCI	13	90–10	88.72
Proposed approach using multi-cumulants and evolutionary hybrid classifier (with ROST balancing)		MLII UCI PTBDB	17 13 2	50–50	100 100 99.43
Proposed approach using multi-cumulants and evolutionary hybrid classifier (with IRST balancing)		MLII UCI PTBDB	17 13 2	90–10	98.04 89.74 99.24

*KNN* K nearest neighbour, *RBF* radial basis function, SVM support vector machine, *KICA + LIBSVM* kernel independent component analysis + library for SVM, *CNN* convolutional neural network, *PCAnet* principal component analysis network, *LSTM* long short-term memory, *GWMD-DE* Gabor wavelet multi-linear discriminant based data extraction, *WT-HMM* wavelet transform-hidden Markov model, *DEA-ELM* extreme learning machine using differential evolution algorithm.

On MLII ECG database, 100% accuracy is obtained with the proposed approach of evolutionary hybrid classifier with multi-cumulants as feature extraction step. Proposed method outperforms the other state-of-art approaches viz. KICA + LIBSVM^88^, 1-D CNN^65^, PCAnet + SVM^89^, CNN + LSTM^90^, Evolutionary-Neural System based on SVM^64^, WT-HMM model ^91^, Ensemble SVM^92^. All these approaches used MLII database with 4 or 5 classes. They have merged the samples depending upon their category of cardiac disorders^88–92^. Only^64,65^ have utilized the MLII database with 17 classes and achieved the accuracies of 90% and 91.33% respectively. They have obtained these results with Train-Test ratio 70–30. However, in our proposed approach, 100% accuracy is achieved with MLII database (with ROST balancing) with 50–50 Train-Test ratio. It also signifies that with lesser amount of training data only, excellent results are obtained. If MLII database (with IRST balancing) is considered, then also, 96.41% accuracy (Table 7) is achieved with 70–30 Train-Test ratio with the proposed approach which is also better than the existing state-of-art approaches.

On UCI repository arrhythmia database as well, results of various existing techniques are compared with the proposed approach. Existing approaches like Kernel Difference Weighted KNN^93^, Modular Neural Network^81^, Wrapper method^97^, Kernel Extreme Learning Machine^67^, GWMD-DE technique^59^, KELM with GA^66^, Ensemble SVM with GA-SVM features selection ^101^ have achieved quite promising results over this database as shown in Table 10 except GWMD-DE technique^59^. In^59^, With 50–50 Train-Test ratio only, they have achieved 96% of accuracy in classifying the ECG signals of UCI arrhythmia database. With the proposed approach, we have attained 100% accuracy on this database as well with 30–70 or more Train-Test ratio as shown in Table 8. This result is using ROST for balancing of UCI database. With IRST as balancing technique, proposed approach outperforms the results of all other techniques in Table 10 except ^59^ as 80.19% accuracy is achieved with 50–50 Train-Test ratio over this database.

At last, proposed approach is compared on PTBDB ECG database having 13 classes with some of the latest state-of-art works on ECG classification. These includes Naïve Bayes^94^, RBF SVM^95^, Convolutional Neural Network^96^, Third-order tensor based analysis^50^, Deep Neural Network^98^, CNN with and without feature extraction^57^, Wavelet KELM^99^, DEA-ELM^100^. All the researchers have considered 2 classes in PTBDB ECG classes viz. normal ECG and abnormal ECG (i.e. with some cardiac disorder). Proposed approach outperforms all these techniques by achieving 99.43% of accuracy is classifying the ECG signals.

The reason behind achieving excellent results for the proposed approach is the use of pre-processing and feature extraction steps before classification. All the three databases of ECG utilized during experimentation face the problem of imbalance (Table 6) which is overcome by ROST and IRST. Noise removal is performed using Daubechies (db6) with level of decomposition 10. Features are extracted in terms of statistical parameters using higher order cumulants. In the classification step as well, evolutionary hybrid classifier optimizes the various parameters to achieve preciseness in the results using the proposed approach.

### Conclusion and future scope

For the classification of ECG signals, a novel and robust approach has been introduced. ECG signals refinement using pre-processing techniques and feature extraction using statistical measures is proved to be a precise and efficient approach for classification of ECG signals. The proposed approach provides excellent results irrespective of signal type whether complete ECG signals, parameters obtained from signals based ECG data or down-sampled ECG signals. Use of evolutionary hybrid classifier also helps in computing the results more precisely. This proposed approach based on feature extraction using multi-cumulants gives 100% accurate results for complete ECG signals of MLII and UCI repository arrhythmia database in which data is parameters obtained from ECG signals. The results obtained on PTBDB database are also very good with maximum percentage accuracy achieved are 99.24% (with IRST) and 99.57% (with ROST). Even the use of ROS techniques for balancing of database makes the processing slow by increasing the execution as the size of database becomes very large. Here, this problem of speed is compensated using the non-iterative classifier (KELM). The results obtained are better than the existing state-of-art approaches as it is already shown in previous section.

As future work, the proposed method can be tested over some live ECG databases. More resampling techniques can further be tested for data balancing as the size of database becomes very large in ROST and creates an issue of slow processing. More refinement can be done in the down-sampled ECG database like PTBDDB database to achieve more accurate results.

## References

[1] Wiggins M, Saad A, Litt B, Vachtsevanos G (2008). Evolving a Bayesian classifier for ECG-based age classification in medical applications. Appl. Soft Comput..

[2] Karlen W, Mattiussi C, Floreano D (2009). Sleep and wake classification with ECG and respiratory effort signals. IEEE Trans. Biomed. Circuits Syst..

[3] Oresko JJ (2010). A wearable smartphone-based platform for real-time cardiovascular disease detection via electrocardiogram processing. IEEE Trans. Inf. Technol. Biomed..

[4] Odinaka I (2012). ECG biometric recognition: A comparative analysis. IEEE Trans. Inf. Forensics Secur..

[5] Sornmo, L., Borjesson, P. O., Nygards, M.-E. & Pahlm, O. A method for evaluation of QRS shape features using a mathematical model for the ECG. *IEEE Trans. Biomed. Eng.* 713–717 (1981). 10.1109/TBME.1981.324666 7319506

[6] Takagi, T. & Sugeno, M. Fuzzy identification of systems and its applications to modeling and control. *IEEE Trans. Syst. Man. Cybern.* 116–132 (1985).

[7] Koski A (1996). Modelling ECG signals with hidden Markov models. Artif. Intell. Med..

[8] Bortolan G, Brohet C, Fusaro S (1996). Possibilities of using neural networks for ECG classification. J. Electrocardiol..

[9] Dokur Z, Ölmez T, Yazgan E, Ersoy OK (1997). Detection of ECG waveforms by neural networks. Med. Eng. Phys..

[10] de Azevedo Botter, E., Nascimento, C. L. & Yoneyama, T. A neural network with asymmetric basis functions for feature extraction of ECG P waves. *IEEE Trans. Neural Netw.* **12**, 1252–1255 (2001). 10.1109/72.950154 18249952

[11] Stamkopoulos T, Diamantaras K, Maglaveras N, Strintzis M (1998). ECG analysis using nonlinear PCA neural networks for ischemia detection. IEEE Trans. Signal Process..

[12] Dokur Z, Olmez T, Yazgan E (1999). Comparison of discrete wavelet and Fourier transforms for ECG beat classification. Electron. Lett..

[13] Kundu M, Nasipuri M, Basu DK (2000). Knowledge-based ECG interpretation: A critical review. Pattern Recognit..

[14] Özbay Y, Ceylan R, Karlik B (2011). Integration of type-2 fuzzy clustering and wavelet transform in a neural network based ECG classifier. Expert Syst. Appl..

[15] Biel L, Pettersson O, Philipson L, Wide P (2001). ECG analysis: A new approach in human identification. IEEE Trans. Instrum. Meas..

[16] Owis MI, Abou-Zied AH, Youssef A-B, Kadah YM (2002). Study of features based on nonlinear dynamical modeling in ECG arrhythmia detection and classification. IEEE Trans. Biomed. Eng..

[17] Blanco-Velasco M, Cruz-Roldán F, López-Ferreras F, Bravo-Santos A, Martinez-Munoz D (2004). A low computational complexity algorithm for ECG signal compression. Med. Eng. Phys..

[18] Tsipouras MG, Fotiadis DI, Sideris D (2005). An arrhythmia classification system based on the RR-interval signal. Artif. Intell. Med..

[19] Israel SA, Irvine JM, Cheng A, Wiederhold MD, Wiederhold BK (2005). ECG to identify individuals. Pattern Recognit..

[20] Mitra S, Mitra M, Chaudhuri BB (2006). A rough-set-based inference engine for ECG classification. IEEE Trans. Instrum. Meas..

[21] Singh BN, Tiwari AK (2006). Optimal selection of wavelet basis function applied to ECG signal denoising. Digit. Signal Process..

[22] Samet H (2008). K-nearest neighbor finding using MaxNearestDist. IEEE Trans. Pattern Anal. Mach. Intell..

[23] Vishwakarma VP, Dalal S (2020). A novel non-linear modifier for adaptive illumination normalization for robust face recognition. Multimed. Tools Appl..

[24] Christov I (2006). Comparative study of morphological and time-frequency ECG descriptors for heartbeat classification. Med. Eng. Phys..

[25] Meau YP, Ibrahim F, Narainasamy SAL, Omar R (2006). Intelligent classification of electrocardiogram (ECG) signal using extended Kalman Filter (EKF) based neuro fuzzy system. Comput. Methods Programs Biomed..

[26] Übeyli ED (2007). ECG beats classification using multiclass support vector machines with error correcting output codes. Digit. Signal Process..

[27] Yu S-N, Chou K-T (2007). A switchable scheme for ECG beat classification based on independent component analysis. Expert Syst. Appl..

[28] Yu S-N, Chou K-T (2008). Integration of independent component analysis and neural networks for ECG beat classification. Expert Syst. Appl..

[29] Ye C, Kumar BVKV, Coimbra MT (2012). Heartbeat classification using morphological and dynamic features of ECG signals. IEEE Trans. Biomed. Eng..

[30] Kampouraki A, Manis G, Nikou C (2008). Heartbeat time series classification with support vector machines. IEEE Trans. Inf. Technol. Biomed..

[31] Khazaee A, Ebrahimzadeh A (2010). Classification of electrocardiogram signals with support vector machines and genetic algorithms using power spectral features. Biomed. Signal Process. Control.

[32] Khazaee A, Zadeh AE (2014). ECG beat classification using particle swarm optimization and support vector machine. Front. Comput. Sci..

[33] Ince T, Kiranyaz S, Gabbouj M (2009). A generic and robust system for automated patient-specific classification of ECG signals. IEEE Trans. Biomed. Eng..

[34] Wang J-S, Chiang W-C, Hsu Y-L, Yang Y-TC (2013). ECG arrhythmia classification using a probabilistic neural network with a feature reduction method. Neurocomputing.

[35] Xie B, Minn H (2012). Real-time sleep apnea detection by classifier combination. IEEE Trans. Inf. Technol. Biomed..

[36] Rai HM, Trivedi A, Chatterjee K, Shukla S (2014). R-peak detection using daubechies wavelet and ECG signal classification using radial basis function neural network. J. Inst. Eng. Ser. B.

[37] Khorrami H, Moavenian M (2010). A comparative study of DWT, CWT and DCT transformations in ECG arrhythmias classification. Expert Syst. Appl..

[38] Özbay Y, Tezel G (2010). A new method for classification of ECG arrhythmias using neural network with adaptive activation function. Digit. Signal Process..

[39] Ceylan R, Özbay Y, Karlik B (2014). comparison of type-2 fuzzy clustering-based cascade classifier models for ECG arrhythmias. Biomed. Eng. Appl. Basis Commun..

[40] Kamath C (2011). ECG beat classification using features extracted from Teager energy functions in time and frequency domains. IET Signal Process..

[41] Xianhai G (2011). Study of emotion recognition based on electrocardiogram and RBF neural network. Proc. Eng..

[42] Muthuvel, K., Suresh, L. P., Alexander, T. J. & Veni, S. H. K. Classification of ECG signal using hybrid feature extraction and neural network classifier. in *Power Electronics and Renewable Energy Systems* 1537–1544 (Springer, 2015).

[43] Alickovic, E. & Subasi, A. Effect of multiscale PCA de-noising in ECG beat classification for diagnosis of cardiovascular diseases. *Circuits Syst. Signal Process.* **34**, 513–533 (2015).

[44] Alickovic E, Subasi A (2016). Medical decision support system for diagnosis of heart arrhythmia using DWT and random forests classifier. J. Med. Syst..

[45] Li, H. *et al.* A new ECG signal classification based on WPD and ApEn feature extraction. *Circuits, Syst. Signal Process.* **35**, 339–352 (2016).

[46] Li H, Yuan D, Ma X, Cui D, Cao L (2017). Genetic algorithm for the optimization of features and neural networks in ECG signals classification. Sci. Rep..

[47] Kora P, Krishna KSR (2016). ECG based heart arrhythmia detection using wavelet coherence and bat algorithm. Sens. Imaging.

[48] Dalal S, Birok R (2016). Analysis of ECG signals using hybrid classifier. Int. Adv. Res. J. Sci. Eng. Technol..

[49] Haldar NAH, Khan FA, Ali A, Abbas H (2017). Arrhythmia classification using Mahalanobis distance based improved Fuzzy C-means clustering for mobile health monitoring systems. Neurocomputing.

[50] Padhy S, Dandapat S (2017). Third-order tensor based analysis of multilead ECG for classification of myocardial infarction. Biomed. Signal Process. Control.

[51] Xu SS, Mak M-W, Cheung C-C (2018). Towards end-to-end ECG classification with raw signal extraction and deep neural networks. IEEE J. Biomed. Health Inform..

[52] Pourbabaee, B., Roshtkhari, M. J. & Khorasani, K. Deep convolutional neural networks and learning ECG features for screening paroxysmal atrial fibrillation patients. *IEEE Trans. Syst. Man, Cybern. Syst.* **48**, 2095–2104 (2018).

[53] Kiranyaz S, Ince T, Gabbouj M (2015). Real-time patient-specific ECG classification by 1-D convolutional neural networks. IEEE Trans. Biomed. Eng..

[54] Xia Y (2018). An automatic cardiac arrhythmia classification system with wearable electrocardiogram. IEEE Access.

[55] Li F (2019). Feature extraction and classification of heart sound using 1D convolutional neural networks. EURASIP J. Adv. Signal Process..

[56] DALAL, S. *A Comparative Study and Analysis on the Classification of ECG Signals*. (Thesis, Delhi Technological University, 2016).

[57] Hammad M, Zhang S, Wang K (2019). A novel two-dimensional ECG feature extraction and classification algorithm based on convolution neural network for human authentication. Futur. Gener. Comput. Syst..

[58] Marinho LB (2019). A novel electrocardiogram feature extraction approach for cardiac arrhythmia classification. Futur. Gener. Comput. Syst..

[59] Velmurugan S, Basha AM, Vijayakumar M (2019). Gabor wavelet multi-linear discriminant analysis for data extraction in ECG signals. Cluster Comput..

[60] Lichman, M. UCI repository arrhythmia database.in *{UCI} Machine Learning Repository*. https://archive.ics.uci.edu/ml/datasets/Arrhythmia (2013)

[61] Cirrincione, G., Randazzo, V. & Pasero, E. A neural based comparative analysis for feature extraction from ECG signals. in *Neural Approaches to Dynamics of Signal Exchanges* 247–256 (Springer, 2020).

[62] Jha, C. K. & Kolekar, M. H. Cardiac arrhythmia classification using tunable Q-wavelet transform based features and support vector machine classifier. *Biomed. Signal Process. Control* **59**, 101875 (2020).

[63] Qaisar SM, Subasi A (2020). Cloud-based ECG monitoring using event-driven ECG acquisition and machine learning techniques. Phys. Eng. Sci. Med..

[64] Pławiak P (2018). Novel methodology of cardiac health recognition based on ECG signals and evolutionary-neural system. Expert Syst. Appl..

[65] Yildirim, Ö., Pławiak Pawełand Tan, R.-S. & Acharya, U. R. Arrhythmia detection using deep convolutional neural network with long duration ECG signals. *Comput. Biol. Med.* **102**, 411–420 (2018). 10.1016/j.compbiomed.2018.09.009 30245122

[66] Dalal S, Vishwakarma VP (2020). GA based KELM optimization for ECG classification. Proc. Comput. Sci..

[67] Dalal, S., Vishwakarma, V. P. & Sisaudia, V. ECG classification using kernel extreme learning machine. in *2nd IEEE International Conference on Power Electronics, Intelligent Control and Energy systems (ICPEICES-2018)* 988–992. 10.1109/ICPEICES.2018.8897416 (2018).

[68] Bhatia, A., Chug, A. & Singh, A. P. Hybrid SVM-LR classifier for powdery mildew disease prediction in tomato plant. in *2020 7th International Conference on Signal Processing and Integrated Networks (SPIN)* 218–223 (2020).

[69] Papoulis, A. & Pillai, S. U. *Probability, Random Variables, and Stochastic Processes*. (Tata McGraw-Hill Education, 2002).

[70] Sharmila, V., HariKrishna, E., Reddy, K. N. & Reddy, K. A. A new method for enhancement of ECG signals using cumulant based AR modeling. in *2013 IEEE Conference on Information & Communication Technologies* 634–637 (2013).

[71] Huang G-B, Zhu Q-Y, Siew C-K (2006). Extreme learning machine: theory and applications. Neurocomputing.

[72] Huang, G.-B., Zhu, Q.-Y. & Siew, C.-K. Extreme learning machine: A new learning scheme of feedforward neural networks. in *Proceedings of the 2004 IEEE International Joint Conference on Neural Networks, 2004. *Vol. 2, 985–990 (2004).

[73] Vishwakarma, V. P. & Dalal, S. A novel approach for compensation of light variation effects with KELM classification for efficient face recognition. in *International Conference on VLSI, Communication and Signal Processing (VCAS 2018)* (2018).

[74] Dalal S, Vishwakarma VP (2020). A novel approach of face recognition using optimized adaptive illumination-normalization and KELM. Arab. J. Sci. Eng..

[75] Dalal, S. & Vishwakarma, V. P. PHT and KELM based face recognition. in *Modern Approaches in Machine Learning and Cognitive Science: A Walkthrough* 157–167 (Springer, 2020).

[76] Vishwakarma, V. P. & Dalal, S. Neuro-fuzzy hybridization using modified S membership function and kernel extreme learning machine for robust face recognition under varying illuminations. *EAI Endorsed Trans. Scalable Inf. Syst. Online First* 1–11. 10.4108/eai.13-7-2018.163575 (2020).

[77] Huang, G. Bin & Chen, L. Enhanced random search based incremental extreme learning machine. *Neurocomputing* **71**, 3460–3468 (2008).

[78] Huang, G.-B., Zhou, H., Ding, X. & Zhang, R. Extreme learning machine for regression and multiclass classification. *IEEE Trans. Syst. Man, Cybern. Part B* **42**, 513–529 (2012). 10.1109/TSMCB.2011.2168604 21984515

[79] Gonzalez, R. C., Woods, R. E. & Masters, B. R. Digital image processing, third edition. *J. Biomed. Opt.* **14**, 029901 (2008).

[80] Goldberger AL (2000). PhysioBank, PhysioToolkit, and PhysioNet: components of a new research resource for complex physiologic signals. Circulation.

[81] Jadhav SM, Nalbalwar SL, Ghatol AA (2011). Modular neural network based arrhythmia classification system using ECG signal data. Int. J. Inf. Technol. Knowl. Manag..

[82] Polat, K. & Günecs, S. Detection of ECG Arrhythmia using a differential expert system approach based on principal component analysis and least square support vector machine. *Appl. Math. Comput.* **186**, 898–906 (2007).

[83] *The PTB Diagnostic Database*. https://www.physionet.org/physiobank/database/ptbd. 10.13026/C28C71.

[84] Pan, J. & Tompkins, W. J. A real-time QRS detection algorithm. *IEEE Trans. Biomed. Eng.* 230–236 (1985). 10.1109/TBME.1985.325532 3997178

[85] Rai HM, Trivedi A (2012). De-noising of ECG waveforms based on multi-resolution wavelet transform. Int. J. Comput. Appl..

[86] Banerjee S, Gupta R, Mitra M (2012). Delineation of ECG characteristic features using multiresolution wavelet analysis method. Measurement.

[87] Ikelle, L. T. & Amundsen, L. *Introduction to Petroleum Seismology*. (Society of Exploration Geophysicists, 2018).

[88] Li, H. *et al.* Novel ECG signal classification based on KICA nonlinear feature extraction. *Circuits Syst. Signal Process.* **35**, 1187–1197 (2016).

[89] Yang W, Si Y, Wang D, Guo B (2018). Automatic recognition of arrhythmia based on principal component analysis network and linear support vector machine. Comput. Biol. Med..

[90] Oh, S. L., Ng, E. Y. K., San Tan, R. & Acharya, U. R. Automated diagnosis of arrhythmia using combination of CNN and LSTM techniques with variable length heart beats. *Comput. Biol. Med.* **102**, 278–287 (2018). 10.1016/j.compbiomed.2018.06.002 29903630

[91] Sangaiah, A. K., Arumugam, M. & Bian, G.-B. An intelligent learning approach for improving ECG signal classification and arrhythmia analysis. *Artif. Intell. Med.* **103**, 101788 (2020). 10.1016/j.artmed.2019.101788 32143795

[92] Pandey SK, Janghel RR, Vani V (2020). Patient specific machine learning models for ECG signal classification. Proc. Comput. Sci..

[93] Zuo WM, Lu WG, Wang KQ, Zhang H (2008). Diagnosis of cardiac arrhythmia using kernel difference weighted KNN classifier. Comput. Cardiol..

[94] Safdarian N, Dabanloo NJ, Attarodi G (2014). A new pattern recognition method for detection and localization of myocardial infarction using T-wave integral and total integral as extracted features from one cycle of ECG signal. J. Biomed. Sci. Eng..

[95] Sharma LN, Tripathy RK, Dandapat S (2015). Multiscale energy and eigenspace approach to detection and localization of myocardial infarction. IEEE Trans. Biomed. Eng..

[96] Acharya UR (2017). Application of deep convolutional neural network for automated detection of myocardial infarction using ECG signals. Inf. Sci. (Ny).

[97] Mustaqeem, A., Anwar, S. M., Majid, M. & Khan, A. R. Wrapper method for feature selection to classify cardiac arrhythmia. in *Engineering in Medicine and Biology Society (EMBC), 2017 39th Annual International Conference of the IEEE* 3656–3659 (2017). 10.1109/EMBC.2017.8037650 29060691

[98] Kachuee, M., Fazeli, S. & Sarrafzadeh, M. Ecg heartbeat classification: A deep transferable representation. in *2018 IEEE International Conference on Healthcare Informatics (ICHI)* 443–444 (2018).

[99] Diker A, Avci D, Avci E, Gedikpinar M (2019). A new technique for ECG signal classification genetic algorithm wavelet kernel extreme learning machine. Optik (Stuttg)..

[100] Diker, A., Avci, E., Tanyildizi, E. & Gedikpinar, M. A novel ECG signal classification method using DEA-ELM. *Med. Hypotheses* **136**, 109515 (2020). 10.1016/j.mehy.2019.109515 31855682

[101] Kadam V, Jadhav S, Yadav S (2020). Bagging based ensemble of support vector machines with improved elitist GA-SVM features selection for cardiac arrhythmia classification. Int. J. Hybrid Intell. Syst..

